# Structural basis for a conserved neutralization epitope on the receptor-binding domain of SARS-CoV-2

**DOI:** 10.1038/s41467-023-35949-8

**Published:** 2023-01-19

**Authors:** Kuan-Ying A. Huang, Xiaorui Chen, Arpita Mohapatra, Hong Thuy Vy Nguyen, Lisa Schimanski, Tiong Kit Tan, Pramila Rijal, Susan K. Vester, Rory A. Hills, Mark Howarth, Jennifer R. Keeffe, Alexander A. Cohen, Leesa M. Kakutani, Yi-Min Wu, Md Shahed-Al-Mahmud, Yu-Chi Chou, Pamela J. Bjorkman, Alain R. Townsend, Che Ma

**Affiliations:** 1 Graduate Institute of Immunology and Department of Pediatrics, National Taiwan University Hospital, College of Medicine, National Taiwan University, Taipei, Taiwan; 2 Genomics Research Center, Academia Sinica, Taipei, Taiwan; 3 College of Medicine, Chang Gung University, Taoyuan, Taiwan; 4 Chemical Biology and Molecular Biophysics program, Taiwan International Graduate Program, Academia Sinica, Taipei, Taiwan; 5 Institute of Biochemical Sciences, National Taiwan University, Taipei, Taiwan; 6 MRC Human Immunology Unit, Weatherall Institute of Molecular Medicine, University of Oxford, John Radcliffe Hospital, Oxford, UK; 7 Department of Biochemistry, University of Oxford, Oxford, OX1 3QU UK; 8 Division of Biology and Biological Engineering, California Institute of Technology, Pasadena, USA; 9 Institute of Biological Chemistry, Academia Sinica, Taipei, 11529 Taiwan; 10 Biomedical Translation Research Center, Academia Sinica, Taipei, 11529 Taiwan; 11 Present Address: Department of Pharmacology, University of Cambridge, Tennis Court Road, Cambridge, CB2 1PD UK

**Keywords:** Antibodies, SARS-CoV-2

## Abstract

Antibody-mediated immunity plays a crucial role in protection against SARS-CoV-2 infection. We isolated a panel of neutralizing anti-receptor-binding domain (RBD) antibodies elicited upon natural infection and vaccination and showed that they recognize an immunogenic patch on the internal surface of the core RBD, which faces inwards and is hidden in the “down” state. These antibodies broadly neutralize wild type (Wuhan-Hu-1) SARS-CoV-2, Beta and Delta variants and some are effective against other sarbecoviruses. We observed a continuum of partially overlapping antibody epitopes from lower to upper part of the inner face of the RBD and some antibodies extend towards the receptor-binding motif. The majority of antibodies are substantially compromised by three mutational hotspots (S371L/F, S373P and S375F) in the lower part of the Omicron BA.1, BA.2 and BA.4/5 RBD. By contrast, antibody IY-2A induces a partial unfolding of this variable region and interacts with a conserved conformational epitope to tolerate all antigenic variations and neutralize diverse sarbecoviruses as well. This finding establishes that antibody recognition is not limited to the normal surface structures on the RBD. In conclusion, the delineation of functionally and structurally conserved RBD epitopes highlights potential vaccine and therapeutic candidates for COVID-19.

## Abstract (web summary)

An antibody, IY-2A, identified from a panel of class-4 SARS-CoV-2-neutralizing antibodies isolated from convalescent and vaccinated individuals, targets and induces partial unfolding of a conserved epitope within the RBD. IY-2A retains activity against BA.4/5 subvariants and neutralizes diverse sarbecoviruses.

## Introduction

Severe acute respiratory syndrome coronavirus 2 (SARS-CoV-2) continues to spread and cause outbreaks worldwide. Antibody-mediated immunity established via natural infection or vaccination reduces the risk of disease or lessens the clinical severity of the infection^1,2^. Neutralizing antibody levels and spike-binding antibody levels serve as correlates of protection against SARS-CoV-2 in humans^1,2^. Although neutralizing antibodies are elicited against other parts of the spike protein, the receptor-binding domain (RBD) is the dominant target of the neutralizing antibody response^3–7^.

The antigenic characterization of the RBD by human neutralizing antibodies revealed four major antigenic sites, two of which (class 1 and class 2) are located at the top of RBD and overlap with the ACE2-binding site (or RBM, the receptor-binding motif), while the other two are on the external (class 3) and internal (class 4) surfaces of the core RBD and extend beyond the ACE2-binding region^6,8,9^. The internal and external surfaces are defined as inward-facing (hidden) and outward-facing (exposed) areas of the RBD in the “down” conformation, respectively. Class 3 neutralizing antibodies have been effectively used alone^10,11^ or in therapeutic combinations with class 1 or class 2 neutralizing antibodies^8,12,13^. Class 4 anti-RBD antibodies mainly recognize a patch of the molecular surface conserved between SARS-CoV-2 and SARS-CoV. Class 4 antibody neutralization may be mediated either by destabilization of the spike structure or hindrance of ACE2 access^6,7,9,14^.

We previously isolated and characterized a typical class 4 neutralizing monoclonal antibody (mAb) EY-6A that binds to a highly conserved epitope composed mainly of the 384–389 helix of the RBD core^6,14,15^. We now describe a panel of antibodies that were identified as class 4 by exhibiting complete or partial competition for RBD binding with EY-6A^6,14^ or similar antibodies. Structural analysis of their binding footprints and comparison with examples in the literature help to reveal antigenic regions consisting of multiple overlapping epitopes, one of which is newly defined by our antibody IY-2A. These antibodies tend to bind to highly conserved epitopes and were broadly effective against SARS-CoV-2 variants of concern.

## Results

### Specificity of class 4 anti-RBD antibodies

We isolated eight class 4 anti-RBD antibodies from SARS-CoV-2 convalescent patients or SARS-CoV-2 vaccinated individuals (Table 1). Antibodies were encoded with distinct heavy chain and light chain variable domain rearrangement and harbored an average of 4 ± 1 (mean ± standard error of the mean) somatic amino acid substitutions in the heavy chain variable domain (Table 1 and Table S1).

**Table 1:** Antigenic specificity and cross-reactivity of class 4 anti-SARS-CoV-2 RBD neutralizing human antibodies

mAb^a^	Antigen exposure	Gene usage		SARS-CoV-2^b^			SARS	MERS	OC43	ACE2-blockade^c^
				S	RBD	NTD	S	S	S	
EY-6A^6, 14^	Infection	V_h_3-30	V_K_1-39	1.75	1.81	0.17	1.67	0.16	0.26	+
FP-12A	Infection	V_h_3-30	V_**λ**_6-57	1.76	1.79	0.15	0.12	0.13	0.12	+++
IV-6D	Vaccination	V_h_3-9	V_K_3-15	1.77	1.84	0.11	1.83	0.42	0.77	Neg
IV-4B	Vaccination	V_h_3-9	V_**λ**_1-44	1.84	1.80	0.12	1.24	0.17	0.22	++
IV-10C	Vaccination	V_h_4-39	V_**λ**_6-57	1.82	1.74	0.17	0.25	0.13	0.14	++
IS-9A	Vaccination	V_h_5-10-1	V_K_1-33	1.78	1.88	0.16	1.83	0.14	0.15	+++
IS-11B	Vaccination	V_h_5-10-1	V_K_1-33	1.76	1.75	0.11	1.67	0.15	0.17	+++
IY-2A	Vaccination	V_h_4-34	V_**λ**_6-57	1.80	1.87	0.15	1.78	0.16	0.25	+++
Control										
BS-1A		V_h_3-64	V_**λ**_2-11	0.13	0.12	0.14	0.12	0.15	0.16	Neg
FD-11A^6^	Infection	V_h_3-33	V_**λ**_1-40	1.79	1.77	0.21	0.14	0.13	0.15	++
FI-3A^6^	Infection	V_h_3-53	V_K_1-33	1.80	1.78	0.17	0.14	0.13	0.16	+++
Plasma	Infection			1.69	1.74	1.77	1.05	0.92	1.29	

^a^EY-6A and FP-12A were isolated from two COVID-19 adult patients (Wuhan-Hu-1 infection) in the convalescent phase. IV-6D, IV-4B, IV-10C, IS-9A, and IS-11B were isolated from an adult at day 8 after the second dose of mRNA-1273 COVID-19 vaccine. IY-2A was isolated from an adult at day 7 after the second dose of mRNA-1273 COVID-19 vaccine.

^b^A sample (1 µg/ml) was considered positive when the measured extinction is at least 3 times the OD value of the negative control in the ELISA with SARS-CoV-2, SARS, MERS, and OC43 proteins. BS-1A (1 µg/ml) is an anti-influenza H3 human mAb. FD-11A (1 µg/ml) and FI-3A (1 µg/ml) are class 3 and class 1 anti-SARS-CoV-2 RBD human mAbs. A COVID-19 convalescent plasma is also included as a control.

^c^Inhibition activity, +++ ≥75%, ++ 51-74%, + 25-50%, Neg, <25%.

*mAb* monoclonal antibody, *V*_*h*_ variable region heavy chain, *V*_*K*_ variable region kappa chain, *V*_**λ**_ variable region lambda chain, *S* spike, *RBD* receptor-binding domain, *NTD* N-terminal domain, *Neg* negative.

**Fig. 1:**
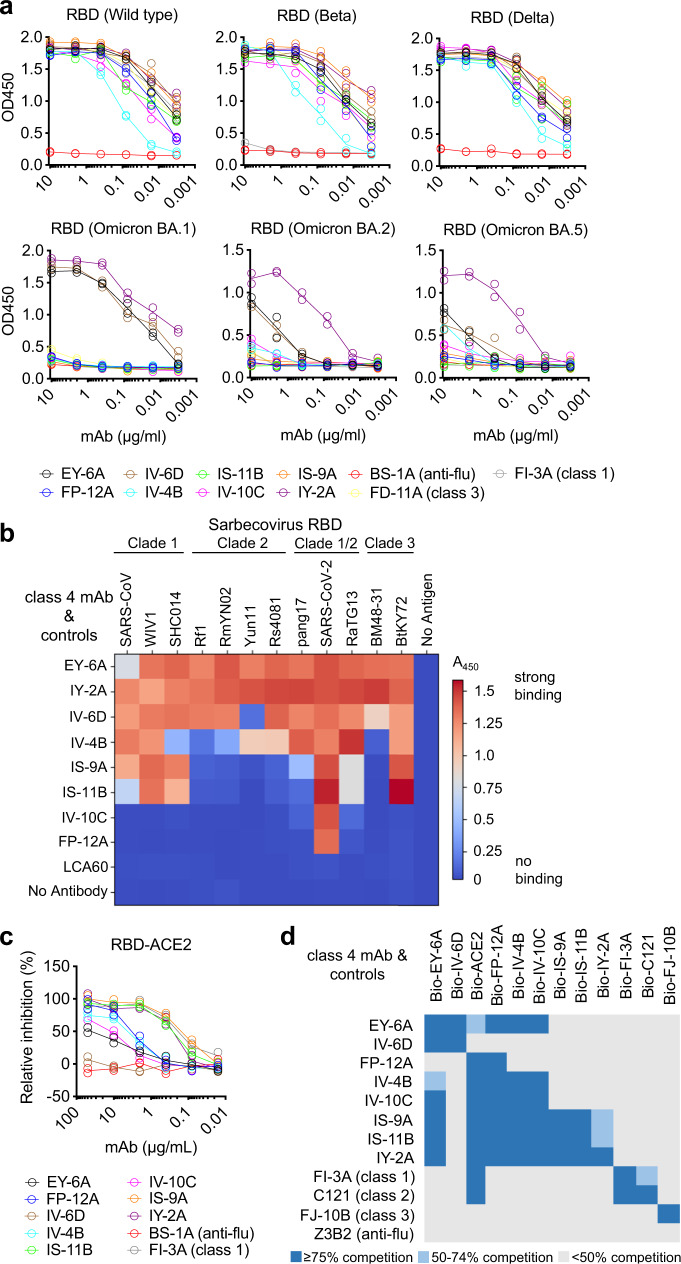
Specificity and epitope grouping of class 4 antibodies. **a** Binding of antibody with RBD of wild type, Beta, Delta, Omicron BA.1, BA.2 and BA.5 variants measured by ELISA. Anti-RBD mAb FD-11 A (class 3) and mAb FI-3A (class 1) were included as controls. Anti-influenza H3 mAb BS-1A was included as a control. OD450, optical density at 450 nm. Each antibody was run with two technical replicates (n = 2) for each RBD antigen. **b** Heat-map showing binding of each antibody (50 nM) to the indicated sarbecovirus RBD, measured by ELISA as OD450 value. Anti-MERS RBD antibody LCA60 was included as a control. **c** The ability of antibody to inhibit binding of the RBD to MDCK-ACE2. Anti-influenza H3 mAb BS-1A was included as a control. Each antibody was run with two technical replicates (*n* = 2) in the experiment. **d** Cross-competition for RBD binding by class 4 antibodies and ACE2. Anti-RBD mAb FI-3A (class 1), mAb C121 (class 2), FJ-10B (class 3), and anti-influenza stem mAb Z3B2 were included as controls. Each antibody was run with four technical replicates (*n* = 4) in the experiment and values are presented as mean. Source data are provided as a Source Data file.

All of the eight antibodies bound to RBD of SARS-CoV-2 spike and cross-reacted with Beta and Delta variant RBDs (Table 1, Fig. 1a). EY-6A^6,14^, IV-6D, and IY-2A retained their binding activity with Omicron BA.1 RBD, but the others lost activity (Fig. 1a, Fig. S1). EY-6A^6,14^ and IV-6D failed to or had greatly reduced activity with BA.2 and BA.5 RBDs, whereas IY-2A binds to all Omicron RBDs in the ELISA (Fig. 1a). Control antibodies FD-11A (class 3) and FI-3A (class 1)^6^ bound strongly to Delta RBD, but both failed to bind all Omicron RBDs (Fig. 1a). Six out of eight antibodies cross-reacted with SARS-CoV spike, but none of the antibodies was reactive to MERS and only one was weakly reactive to OC43 betacoronavirus (Table 1).

There is ~60–90% amino acid identity in the RBD among SARS-CoV-2 and other sarbecoviruses, including SARS-CoV and bat-derived SARS-like viruses^16–18^. We tested the binding of all 8 antibodies against a panel of sarbecovirus RBD from different clades (clade 1; SARS-CoV, WIV1 and SHC014, clade 2; Rf1, RmYN02, Yun11 and Rs4081, clade 1/2; pang17, SARS-CoV-2, and RaTG13, and clade 3; BM48-31 and BtKY72). EY-6A^6,14^ and IY-2A showed binding against the RBD of all sarbecoviruses tested. IV-6D bound to all RBDs except for Yun11 (Fig. 1b). IV-4B, IS-9A, and IS-11B cross-reacted with a limited set of sarbecoviruses, with weak to no binding against clade 2 RBD (Rf1, RmYN02, Yun11) and some clade 1/2 RBD binding (pang17) (Fig. 1b). IV-10C and FP-12A only showed binding to SARS-CoV-2 RBD (Fig. 1b).

The footprint recognized by EY-6A is located at the internal surface of the core RBD^14,19^. EY-6A and another well-characterized antibody CR3022 are designated as class 4 antibodies^5,14,20^. Patterns of cross-competition between these broadly reactive antibodies indicated they recognize a continuum of partially overlapping epitopes of the RBD. In this assay one antibody was labelled with biotin, and binding was measured in conditions of molar excess of the unlabeled competitor. EY-6A^6,14^ and IV-6D competed with each other for binding to RBD and exhibited partial or no interference with the interaction between ACE2 and RBD (Fig. 1c, d). FP-12A showed a one-way pattern of competition with EY-6A but blocked the interaction between ACE2 and RBD (Fig. 1c, d). Five other antibodies competed with EY-6A^6,14^ and exhibited strong ACE2 blocking activities; IV-4B and IV-10C competed with each other, while IS-9A, IS-11B, and IY-2A competed with each other (Fig. 1C, D). IY-2A prevented the binding of IS-9A and IS-11B to RBD, but IS-9A and IS-11B showed slightly less competition with IY-2A. None of these class 4 antibodies competed with the biotinylated FI-3A (class 1)^6^, C121 (class 2)^21^ or FJ-10B (class 3)^6^ binding to the RBD (Fig. 1d).

### Neutralization by class 4 anti-RBD antibodies

**Fig. 2:**
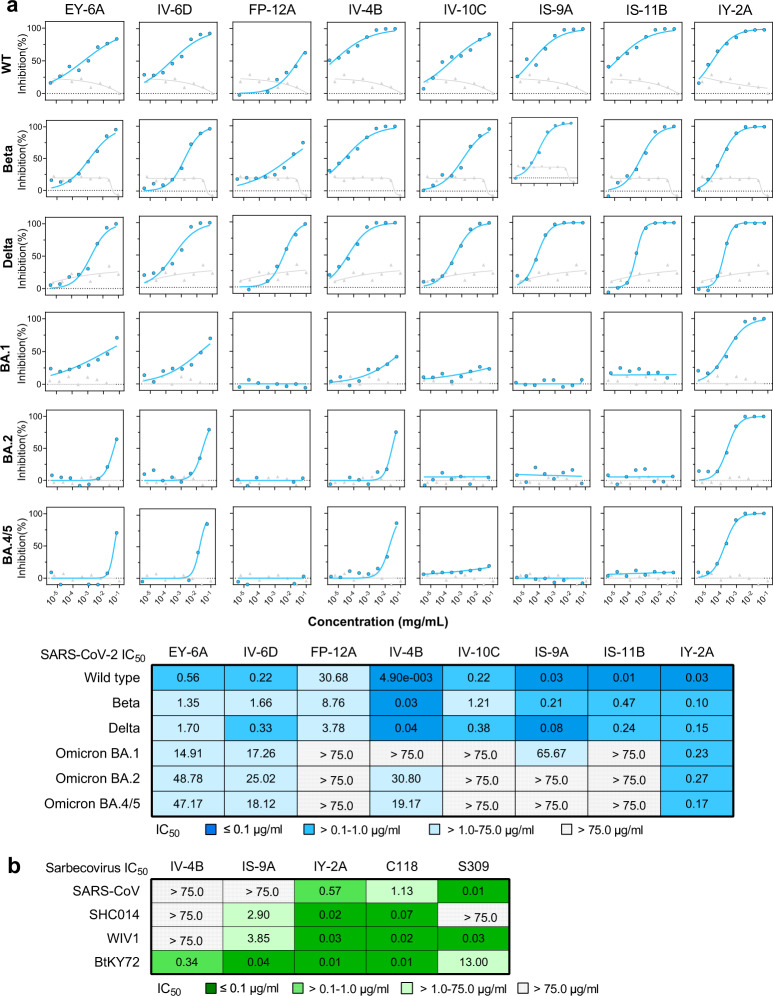
Neutralization breath of class 4 antibodies. **a** Neutralization potency of each antibody against SARS-CoV-2 pseudotyped virus (wild type, Beta, Delta, and Omicron BA.1, BA.2 and BA.4/5 variants). Gray curves are anti-influenza H3 mAb BS-1A as control. Data are mean of technical duplicates (*n* = 2), and curves are fit by nonlinear regression for half-maximal inhibitory concentrations (IC_50_ values), as summarized in the table below. Each box of the table is colored accordingly: the higher the potency, the darker the color. **b** Neutralization potencies of class 4 antibodies using a pseudovirus-based assay of SARS-CoV and sarbecoviruses. C118 (class 4) and S309 (class 3) are anti-RBD mAbs and are included as controls. The IC_50_ values are summarized for each antibody, with each box colored accordingly. Source data are provided as a Source Data file.

All of the class 4 antibodies were assessed for neutralization potency using a SARS-CoV-2 pseudotyped virus assay. The antibodies showed varied levels of neutralizing potency against wild-type (Wuhan-Hu-1) SARS-CoV-2 virus, with IV-4B, IS-9A, IS-11B, and IY-2A being most potent with half-maximal inhibitory concentrations (IC_50_ values) less than 0.1 μg/ml (Fig. 2a). The majority of antibodies exhibited comparable activities against Delta and Beta variants. The superior potencies of IV-4B, IS-9A, IS-11B, and IY-2A against wild type and variants are consistent with their binding and ACE2-blocking activities (Fig. 1a, c, Fig. S1). The majority of class 4 antibodies lost or had greatly reduced neutralization against the Omicron variant. Only IY-2A retained neutralization against Omicron BA.1, BA.2, and BA.4/5, with IC_50_ values of 0.23, 0.27 and 0.17 μg/ml (Fig. 2a), in agreement with its binding activity with Omicron RBDs (Fig. 1a, Fig. S1).

We then tested neutralization potencies of representative antibodies using a pseudovirus-based assay of SARS-CoV and sarbecoviruses known to infect human ACE2-expressing target cells (SHC014 and WIV1) as previously described^17^ and a mutant BtKY72-CoV2 that is able to bind to human ACE2^22^. We found that IY-2A potently neutralizes all four sarbecoviruses including SARS-CoV (IC_50s_ ranging from 7 to 569 ng/ml), similar to the neutralization potency of a previously described human donor-derived antibody^17^, C118 (IC_50_ ranging from 5 to 1,130 ng/ml) (Fig. 2b). IS-9A and EY-6A^6,14^ neutralized BtKY72, SHC014, and WIV1 but failed to neutralize SARS-CoV, whereas IV-4B only neutralized BtKY72 (Fig. 2b). The control class 3 antibody S309^10^ neutralized SARS-CoV, BtYK72 and WIV1 but failed to neutralize SHC014 as previously described^17^.

### Class 4 RBD epitope-specific serological response

To examine the level of antibodies competing with representative class 4 antibodies in Wuhan strain infection- and vaccine-elicited sera, serum blockade of RBD binding experiments were performed. The results showed that a relatively small percentage of convalescent and post-ChAdOx1 nCoV-19 (AZD1222)^23^ sera demonstrated competitive activity with EY-6A binding, compared to that of post-mRNA-1273 (Moderna COVID-19 vaccine)^24^ sera (Fig. S2a). Similar results were found in the convalescent and post-ChAdOx1 nCoV-19 (AZD1222) sera which competed with IS-9A and IY-2A for RBD binding. Two post-mRNA-1273 sera (2 of 8, 25%) did not compete with IS-9A and marginally competed with IY-2A for RBD binding (Fig. S2b, c). These results suggested that the mRNA-1273 vaccine would likely elicit more class 4 anti-RBD antibodies than the ChAdOx1 nCoV-19 (AZD1222) vaccine.

### Structural features and footprints of class 4 antibodies

**Fig. 3:**
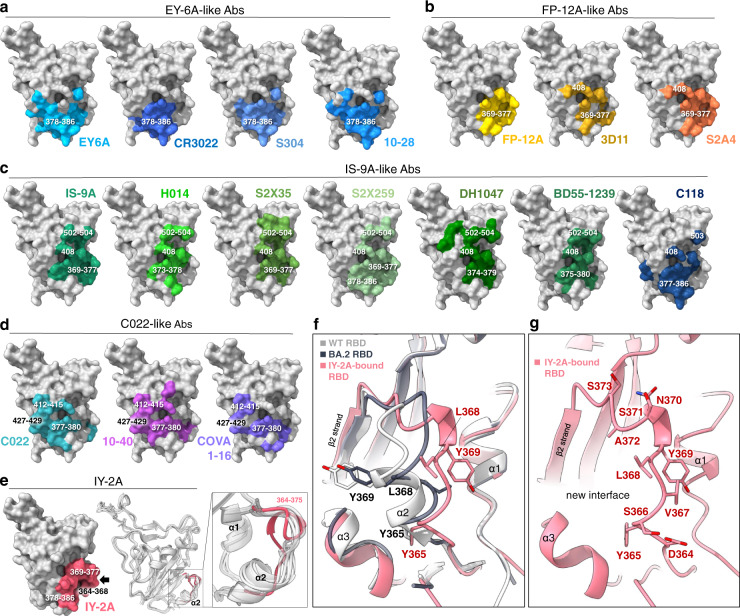
Structural footprints of class 4 antibodies on the RBD and an induced fit of RBD by IY-2A. Footprints of each class 4 antibody on the internal surface of RBD are shown. The footprint includes all the residues that are directly involved in hydrogen-bond (2.5-3.5 Å), salt-bridge (<4 Å), or hydrophobic (3.3-4.0 Å) interaction in the structure. **a** EY-6A^6, 14^ and similar antibodies share the binding site around the residues 378-386 region. PDB code 6ZER for EY-6A, 6W41 for CR3022, 7R6X for S304 and 7SI2 for 10–28. **b** FP-12A and similar antibodies share the binding site around the residues 369-377 region, with some reaching residue 408. PDB code 7M7B for 3D11 and 7JVA for S2A4. **c** IS-9A and similar antibodies extend their footprints upwards and contact residue 408 and the residues 502-504 region. PDB code 7CAH for H014, 7R6W for S2X35, 7M7W for S2X259, 7LD1 for DH1047, 7WRL for BD55-1239 and 7RKS for C118. **d** C022 and similar antibodies extend their footprints towards the left side, including residues 412–415 and 427–429. PDB code 7RKU for C022, 7SD5 for 10–40 and 7JMW for COVA1-16. **e** IY-2A recognizes a region (the 365-369 helix, black arrow), which is originally buried but is now exposed after a conformational change (enlarged view, the backbone shift highlighted in red). Structures (PDB code 6ZER, 7M7W, 7R6X, 7R6W, 6W41, light gray) are used for superimposition. **f** Superimposition of the ACE2-bound wild-type RBD (PDB 6M0J, white). The ACE2-bound Omicron BA.2 RBD (PDB 7ZF7, gray) with IY-2A-bound RBD (red) reveals a rotated view of the conformational change, highlighting residues Y365, L368, and Y369. **g** Detailed structure of the shifted 364–373 region in IY-2A-bound RBD, with all affected residues drawn in sticks and labeled.

Crystal structures of the RBD-Fab complexes were determined for FP-12A, IS-9A and IY-2A for characterizing their footprints on RBD (Table S2). An RBD-Fab complex structure has been solved for EY-6A^14^ and was included for comparison. Although most of the class 4 antibodies bind to an overlapping area on the internal surface of the core RBD, they showed variable approach angles (Fig. S2a). Mapping of these footprints on RBD revealed key structural features that are associated with their behaviors in the competition and binding assays (Fig. 1). For EY-6A^6,14^, the footprint is more focused on the C-terminal half of the long linker between β1 and β3 of RBD (residues 378–386), like that of CR3022^25^, S304^26^ and 10–28^27^ (Fig. 3a, Fig. S3b). For FP-12A, the footprint is focused on the N-terminal half of the linker (residues 369–377), like that of 3D11^28^ and S2A4^26^ (Fig. 3b, Fig. S3b). IS-9A and similar antibodies such as C118^17^, H014^29^, S2X35^26^, S2X259^16^, DH1047^30^, and BD55-1239^31^ exhibited more diversified binding modes (Fig. S3a), and their footprints cover the lower part (residues 373-378), the middle part (residue 408), and the upper part (residues 502–504) of the internal RBD surface (Fig. 3c, Fig. S3b). The footprints of C022^17^ and similar antibodies such as 10-40^27^ and COVA1-16^32^ cover the left part of the RBD (residues 412–415 and 427–429) (Fig. 3d, Fig. S3b).

Finally, a unique footprint is observed for IY-2A, which extends further to the right bottom part of the RBD (residues 364–368, Fig. 3e), a region that is buried in the apo RBD but becomes exposed upon interaction with IY-2A (Fig. 3e). The residues 364–375 region of RBD in this complex exhibits an unwound α2 helix and a newly formed left-handed 3_10_-helix at residues 369–372, creating a novel interface on RBD (Fig. 3f, g). Binding to this interface underlies the excellent and broad neutralizing activity that IY-2A exhibited against SARS-CoV-2 variants and other sarbecoviruses (Fig. 2), which outcompetes all the other class 4 antibodies described here.

A linear peptide (residues 369–386) that spans across the lower internal part of RBD is involved in the footprint of all class 4 antibodies (Fig. S3b). This region contains a short β-strand (β2) in the middle, overlapping with two short α-helices (α2 and α3) on each side (Fig. S3b). Two additional regions recognized mainly by IS-9A include the previously identified highly conserved residue R408 in the α4 helix^17^ (Fig. 3c, Fig. S3b), which has since mutated to serine (R408S) in the Omicron BA.2 and BA.4/5 variants, and the 502-504 region, which overlaps with the RBM (Fig. S3c) in the immediate vicinity of mutational hotspots N501Y (Alpha, Beta, Omicron, etc.) and Y505H (Omicron). From surface accessibility and antigenic variation analyses^33^, it can be deduced that IS-9A and similar antibodies exhibit increased neutralization potencies because these additional recognition sites (R408 and residues 502–504) are more exposed (Fig. S3d), but their neutralization breath can be compromised by emerging variants with mutations at or around these sites (Fig. S3c, e). It should also be noted that three mutational hotspots (S371L/F, S373P, and S375F, Fig. S3e) are in the C-terminal half of the lower region (residues 369–377), which could have a larger influence on the activity of FP-12A, IS-9A and similar antibodies than the EY-6A-like antibodies.

### Structural basis for the functional difference between class 4 antibodies

**Fig. 4:**
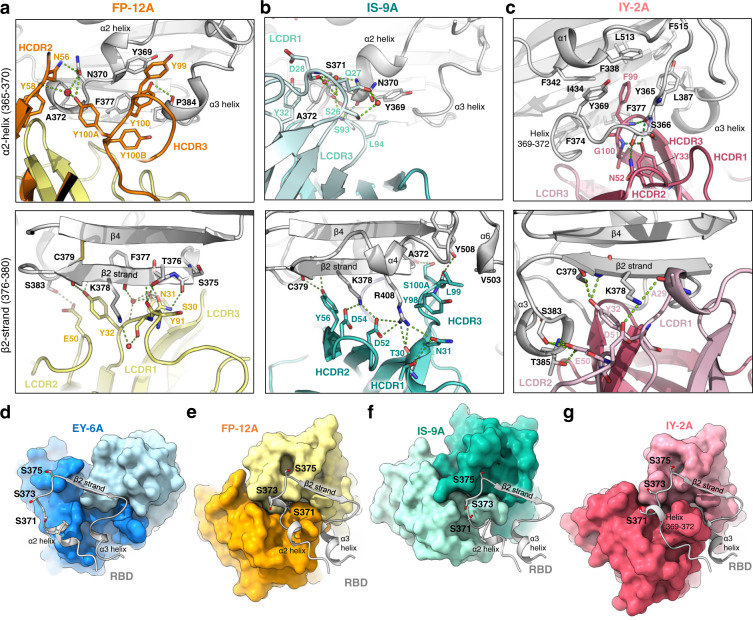
Detailed structural interface between class 4 antibodies and RBD. **a** The interface between FP-12A (heavy chain, orange; light chain, yellow) and two key binding regions on RBD (gray, upper, 365–370 α2 and 384-388 α3 helices; lower, 376–380 β2 strand). Heavy and light chain CDRs (HCDR and LCDR) and key interacting residues are labeled. Hydrogen bonds are shown in green dash lines, and water molecules in red spheres. **b** The same views of interface as in (**a**) between IS-9A (heavy chain, dark teal; light chain, light teal) and RBD (gray). **c** The same views of interface as in (**a**) between IY-2A (heavy chain, dark red; light chain, light red) and RBD (gray). **d**–**g** The binding mode of class 4 antibodies (surface) with the epitope region including α2 helix, β2 strand and α3 helix of RBD (ribbon, in light gray) for EY-6A^6, 14^ (**d** in dark and light blue), FP-12A (**e** in orange and yellow), IS-9A (**f** in dark and light teal) and IY-2A (**g** in dark and light red). Residues of Variants of Concern (S371, S373, and S375) are shown in sticks and labeled. These panels (**d**–**g**) are in exactly the same view.

Structural details of the RBD-mAb interface may aid molecular understanding of their functional differences in neutralization potency and breadth. For example, EY-6A^6,14^ and S304^10^ share an almost identical binding mode with ~91% sequence identity (Fig. S4a), but EY-6A has a stronger hydrophobic interaction at residue W100 (Kabat numbering) of heavy chain complementarity determining region 3 (HCDR3) and residue L95 of light chain complementarity determining region 3 (LCDR3), as well as a strengthened polar contact at residues 52A/53 of HCDR2 (Fig. S4b, c), all possibly contributing to a lower IC_50_ value against SARS-CoV-2 wild type (EY-6A, 0.56 µg/ml; S304, > 2 µg/ml)^6,10,14^. On the other hand, antibody FP-12A is a weaker neutralizer compared to 3D11^28^, although their binding modes almost overlap when superimposed (Fig. S4d). Their detailed structural differences include the Tyr-rich HCDR3 of FP-12A which slightly pushes away the α2 helix, a weaker engagement with the 372–375 region, as well as other residue differences in LCDR1 and 3 (Fig. S4e, Fig. 4a).

Antibody IS-9A and its closest partner, IS-11B, share 94.8% sequence identity in the CDRs (Table S1) and exhibit a slightly higher activity against wild type but lower activity against Beta and Delta variants (Fig. 2a). This difference could be attributed to the substitutions of HCDR1 N31 for S and HCDR3 L99 for Y in IS-11B that modulate the interaction with different antigen variants (Fig. 4b). In IS-9A, the HCDR2 forms a β-turn (sequence D_52_PSD_54_, Kabat numbering) to present two acidic side chains that contact two positively charged RBD residues, K378 and R408 (Fig. 4b, Fig. S5a). R408 is also involved in hydrogen bonds with T30 and N31 from HCDR1 and a cation-π interaction with Y98 from HCDR3 of IS-9A (Fig. 4b, Fig. S5a). This extensive interaction network may be part of the structural basis for IS-9A’s high potency (IC_50_ 0.03 µg/ml), compared to other similar antibodies, while the second potent antibody, S2X259 (IC_50_ 0.14 µg/ml)^16^, employs a 13-residue-long LCDR3 loop (IMGT definition^34^) that closely interacts with RBD’s 501-504 region (Fig. S5b–d).

The IY-2A-RBD structure reveals a novel binding mode for a class 4 antibody (Fig. 4c). IY-2A’s hydrophobic HCDR3 (sequence G_97_IFGV_100A_, Kabat numbering) intrudes deep into the hydrophobic core of RBD, surrounded by seven aromatic residues, two leucines and one isoleucine, including F338, F342, Y365, Y369, F374, F377, F515, L387, L513, and I434 (Fig. 4c). This blade-like HCDR3 is strengthened by the hydroxyl group of Y33 from HCDR1, which forms hydrogen bonds with the backbone nitrogen of F99 and G100 on HCDR3 and the carbonyl oxygen of RBD residue Y365 (Fig. 4c). Such a structure partially refolds the 364–376 region (α2 helix and α2-β2 linker) of RBD, shifts Y369 by up to 7.8 Å (compared to the ACE2-bound RBD, Fig. S6a) and brings S366 into contact with N52 on HCDR2 (Fig. 4c). The observed conformational change is consistent for all four copies in the crystallographic asymmetric unit (Fig. S6a), and the electron density at this region is unambiguous (Fig. S6b). Importantly, this region is known to be structurally conserved and stable among various RBD structures (Fig. S6c, Fig. 3e), but in the recently emerged Omicron BA.1 and BA.2 variants, the region exhibits higher flexibility, yet maintaining an overall similar conformation (Fig. S6d). At least two residues of RBD (Y365 and L368) are brought from the buried to the exposed state (Fig. S6e, Fig. 3f, g), while residues that are found in the interface with other antibodies, such as Y369 and N370 (Fig. 4a, b), become less accessible when IY-2A is bound (Fig. S6e). In addition, the LCDRs of IY-2A interact mostly via hydrogen bonds (e.g., Y32 of LCDR1 and D51 of LCDR2), and unlike other class 4 antibodies, its LCDR3 only has a minimal contact (Fig. 4c).

An opposite direction of view from the RBD side towards the antibody provides a clear structural explanation for the ability of Omicron variants to evade antibody responses (Fig. 2a). While the footprint of EY-6A^6,14^ does not overlap with any of the three hotspot residues (S371, S373, and S375) (Fig. 4d, Fig. S7a), the close engagement of FP-12A (HCDR3 and LCDR3) with S371 (Fig. 4a, e), and of IS-9A (HCDR3) with S375 (Fig. 4b, f), explains their lack of neutralizing activity against Omicron variants (Fig. 2a). The IY-2A footprint also overlaps with these residues (Fig. S7a), but the conformational change induced by IY-2A flips the side-chains of S371, S373, and S375 out of the binding site (Fig. 3e, Fig. 4c, g). Other mutations in Omicron BA.2 and BA.4/5, such as T376A, D405N, and R408S, are not involved in the IY-2A-RBD interface, except that T376 is somewhat close to S30 of LCDR1 (~4 Å) without forming a hydrogen bond (Fig. S6f).

The binding mode of IY-2A is consistent with the finding that it tolerates all the changes in SARS-CoV-2 variants and other sarbecoviruses (Fig. 2). Another antibody that binds widely across sarbecoviruses, EY-6A^6,14^, has reduced RBD binding and neutralization of SARS-CoV (Fig. 1b, Fig. 2b), likely because the S373F substitution alters the local structure and moves the backbone carbonyl groups away from K57 of EY-6A^6,14^ (Fig. S7a, b). On the other hand, the substitution of A372 to T in all sarbecoviruses other than SARS-CoV-2 may have a substantial effect on the FP-12A/RBD interaction (Fig. 1b, Fig. S7a), as RBD residue A372 is tightly packed between the HCDR2 and LCDR3 of FP-12 and there is no space for the side chain of a threonine (Fig. S7c). Similarly, a serine residue at the G404 site of RBD would make an unfavorable hydrophilic contact with L99 of IS-9A (Fig. S7d), thus explaining why IS-9A binds to the RBD of SARS/WIV1/SHC014/RaTG13 but not Rs4081 (Fig. 1b).

### Binding mode of class 4 antibodies to the spike trimer

**Fig. 5:**
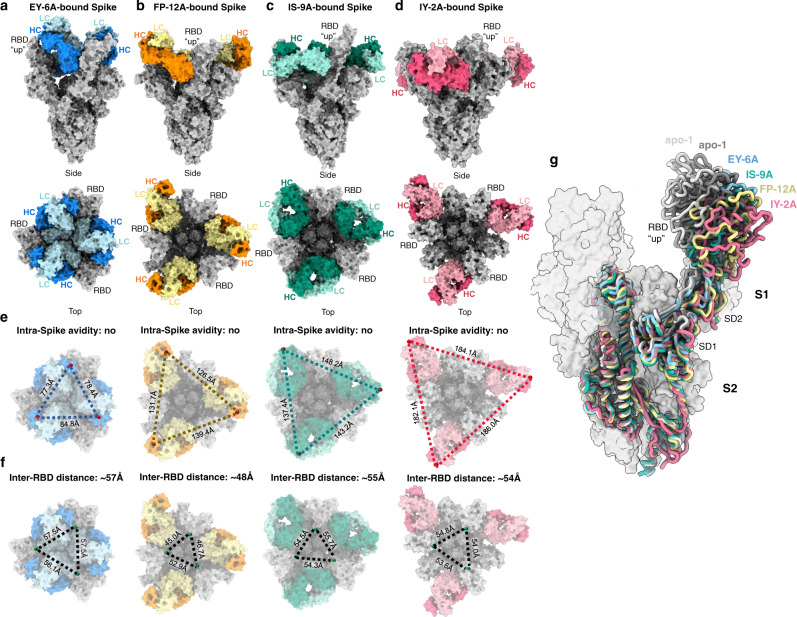
Cryo-EM structures of class-4-mAb-bound Spike protein. **a**–**d** Surface representation of cryo-EM structures of the Spike-Fab complex for EY-6A^6, 14^ (**a**), heavy and light chains in dark and light blue, PDB code 6ZDH), FP-12A (**b**), in orange and yellow), IS-9A (**c**), in dark and light teal) and IY-2A (**d**), in dark and light red). Spike is shown in gray. All bound RBDs are in the “up” conformation. The constant region of Fab is modelled based on EM density at low-threshold rendering. **e** The potential for intra-spike avidity effects (simultaneous binding of both Fabs of a single IgG to adjacent RBDs on a Spike trimer) was evaluated by measuring the distance between the C-terminal (C224) of each bound Fab. Distances are shown in black dashed lines. **f** Inter-RBD distances measured between D428 of RBD for each complex structure. **g** The conformation of RBD of the class-4-mAb-bound Spike compared to that of the apo 2-RBD-up Spike of Delta variant (PDB code 7V7U and 7V7T as representative). Only one subunit of Spike is shown in licorice, with S2 subunit being superimposed. NTDs are omitted for clarity. Both RBD and SD1/2 show visible shifts to further widen the inter-subunit space. Each structure is colored accordingly as in (**a**–**d**). The other two subunits are from IS-9A-bound Spike shown in transparent surface.

The conserved neutralization epitope recognized by class 4 antibodies is known to have low surface accessibility on the intact spike trimer (Fig. S3d), which raises the question as to how these antibodies recognize authentic virus. Our cryo-EM structures of the spike-Fab complexes revealed a three-RBD-up conformation for the complex with all these class 4 antibodies (Fig. 5a–d, Table S3), because only in the open state could their epitopes be accessed. The local footprint differences now result in visibly distinct conformations of the complex (Fig. 5a–d), but none of these antibodies exhibit the possibility of simultaneous binding of both Fabs of an IgG to a single trimer (intra-spike avidity) because of the long distance between the C-termini of adjacent bound Fabs^17^ (Fig. 5e). The Fabs-spike interaction further opens the trimer (Fig. 5f) and tilts the RBD in different degrees compared to the apo structures (Fig. 5g) or the ACE2-bound 3-RBD-up spike, in which the inter-RBD distance is around 36–39 Å.

Generally, the more buried the target region is, the harder for an antibody to approach, but at the same time, the more conserved the interacting residues are (Fig. S3d, e). This is the case for antibody EY-6A^6,14^, which reaches the most conserved 377-386 region and remains broadly interacting (Fig. 1a, b), while antibody IS-9A binds to a more easily accessible area and exhibits a much higher potency but loses activity against Omicron BA.1, BA.2, and BA.4/5 variants (Fig. 1a, Fig. 2a). A more balanced example is shown as antibody IY-2A, which creates a conformational epitope, tolerates all antigenic variations and compensates for the energy cost of its induced fit with extensive hydrophobic interactions (Fig. 4c). The cryo-EM of IY-2A-Spike complex also shows a similar conformation of the 364-375 region with the unwound α2 helix to accommodate the binding of IY-2A (Fig. S8).

## Discussion

There is a need for developing broadly reactive SARS-CoV-2 vaccines and broadly reactive therapeutic mAbs against SARS-CoV-2 variants and animal coronaviruses that have the potential to cause a pandemic. In this study, we identified a cryptic and conserved epitope capable of eliciting broad anti-SARS-CoV-2 antibodies, represented by antibody IY-2A, which has not been reported previously. IY-2A binds not only to diverse SARS-CoV-2 variants but also throughout the sarbecovirus family. Although IY-2A-like antibodies are rare in polyclonal sera (Fig. S2), the high potency and breadth of such antibodies suggest that broad SARS-CoV-2 vaccines could seek to direct responses to this epitope and induce similar broadly neutralizing antibodies. The broad cross-reactive anti-RBD response has been reported from a mosaic RBD nanoparticle vaccine presenting multimerized antigens^35^. Recent clinical trials have also shown a heterologous boost of SARS-CoV-2 vaccine substantially enhances the breadth and potency of anti-RBD antibody response^36–38^, which is associated with improved protection against emerging variants^38,39^. Although a majority of class 4 antibodies did not overcome mutations in the Omicron RBD, the discovery of IY-2A-like antibodies highlights the potential of a potent and cross-reactive anti-RBD antibody response.

The majority of class 4 antibodies reported here, including IY-2A, were derived from peripheral B cell clones elicited upon two doses of SARS-CoV-2 mRNA vaccine (Table 1), which was based on the ancestral Wuhan strain. It has been reported that the neutralizing activity of mRNA vaccine-elicited antibodies was more targeted to the RBD compared to antibodies elicited by natural infection^40^. Moreover, mRNA vaccine-elicited anti-RBD antibodies may recognize more widely distributed epitopes in comparison to infection-elicited antibodies^40,41^. The development of class 4 antibody repertoire indicates that both natural infection and mRNA vaccination elicit antibodies that evolve increased neutralizing activity and breadth. Another study found that broadly neutralizing class 4 antibodies were enriched in SARS convalescents after COVID-19 vaccination^31^. Although the majority of such antibodies are sensitive to D405N and R408S mutations in BA.2 and BA.4/5 variants, a rare antibody BD55-5514 retains neutralization against recently emerged Omicron variants^31^.

Sequence analysis reveals common genetic features shared by anti-RBD human antibodies elicited after vaccination and infection^42^. A preference for V**λ**6-57 light chain gene usage combined with diverse Vh gene usage was observed in a cluster of anti-RBD antibodies^26,42^. FP-12A, IV-10C, and IY-2A share the gene usage of V**λ**6-57 germline. The conserved S_30_NY_32_ motif (Kabat numbering) in CDR1 and E_50_D_51_ motif in CDR2 participate in an extensive hydrogen-bond network with the 376–380 strand and 384–388 helix of RBD (Table S1, Fig. 4). IV-10C was encoded with the Vh4-39 gene which is frequently used in this antibody cluster;^42^ by contrast, each of FP-12A and IY-2A was encoded with its unique heavy chain gene segment and lacks the WLRG motif in HCDR3. EY-6A and IS-9A have distinct genetic usages in the heavy and light chain variable regions and there is no convergence of CDR sequences readily identified in the public anti-RBD antibody responses^42^.

Structural studies of these class 4 antibodies may provide insights about how a single amino acid change, even to a similar-sized side chain, could lead to a major reduction of neutralization potency (Fig. S7). Moreover, the unexpected binding mode of IY-2A has revealed that an epitope of RBD could be created by a conformational change, and in this way the conventional epitope prediction based on the pre-defined structure would be rendered incomplete. However, the neutralization mechanism of our class 4 anti-RBD antibodies against SARS-CoV-2 remains elusive. There are several possibilities. Firstly, we have structurally characterized EY-6A binding in detail to spike by cryo-EM analyses and EY-6A binding would destabilize the native Spike conformation, catalyzing conversion to the post-fusion form^6,14^. EY-6A converts even the stabilized spike to a partially unfolded state, where Fab is visible, still attached to the RBD^6,14^. These findings suggest that neutralization may be mediated by the destruction of the pre-fusion spike^14,43^. Secondly, antibody neutralization may also act by blocking the interaction of viral RBD and receptor ACE2^29,32^. For example, IS-9A, IS-11B, and IY-2A efficiently prevented both the attachment of biotinylated RBD to ACE2-expressing MDCK cells and the binding of biotinylated ACE2 to recombinant RBD (Fig. 1c, d), which could be attributed to steric clashes with ACE2, as observed in superimposed structures (Fig. S9a–c). Similar ACE2-blocking activities were reported in class 4 antibodies^17,27,29,31,32^. Thirdly, it was previously proposed that some class 4 antibodies do not directly compete with ACE2 but may enhance S1 shedding and thus premature spike conformational changes^16,26^, which may offer an alternative mechanism of neutralization.

SARS-CoV-2 is constantly evolving and certain variants of concern have garnered widespread attention because of their rapid emergence within populations^44–46^. In particular, several variants escape antibody immunity and have been reported to be associated with a higher risk of reinfection in individuals previously immunized with a different variant^19,47,48^. RBD plays a substantial role in SARS-CoV-2 antigenicity and the majority of serological neutralizing activity elicited after infection or vaccination is contributed to anti-RBD antibodies^4,25^. Mutations of variants that occur on the RBD, e.g., E484, K417, L452, G446, affect binding and neutralization by polyclonal serum and therapeutic antibodies^9,19,49,50^. Mutations of S371-S373-S375 in the recently emerged Omicron variants escape the majority of class 4 antibodies, as reported in this study. It is difficult to predict which mutations will rise next to prominence as SARS-CoV-2 continues to evolve, but it seems likely that they will include additional RBD mutations that affect recognition by infection- and vaccine-elicited antibodies. The highly conserved 405-408 region was originally retained when Omicron BA.1 first emerged but was quickly mutated (D405N and R408S) and stabilized in BA.2, BA.4, and BA.5 variants in the current worldwide circulation^51^. Therefore, continuous efforts focusing on those broad-based antibody responses, via B cell clonal breadth dissection and high-resolution mapping of antibody epitopes, would help to design an updated vaccine that is maximally cross-reactive to variants and formulate immunization strategy that leads to optimal neutralizing breadth and potency in the near future.

## Methods

### Participants and ethical statement

We enrolled two COVID-19 adult patients (Wuhan-Hu-1 infection)(43 and 55 years old) in the convalescent phase and two adults (26 and 44 years old) after second dose of COVID-19 vaccine. Naturally occurring SARS-CoV-2 infection was diagnosed by positive real-time reverse transcriptase polymerase chain reaction results of respiratory samples according to the guidelines of the Taiwan Centers for Disease Control. The study protocol and informed consent were approved by the ethics committee at the Chang Gung Medical Foundation. Each patient provided signed informed consent. The study and all associated methods were carried out in accordance with the approved protocol, the Declaration of Helsinki and Good Clinical Practice guidelines.

### Isolation of antibodies

SARS-CoV-2 convalescent patients and immunized healthy adults were enrolled and peripheral blood was collected. Peripheral blood mononuclear cells (PBMCs) were prepared from peripheral blood using Ficoll-Paque (Sigma-Aldrich, USA). Single B cells were isolated with or without using biotinylated RBD (Beta variant) into the 96-well PCR plate containing lysis buffer as previously described^6^. After performing reverse transcription to obtain cellular Ig cDNA, variable domain-encoding genes for heavy, kappa and lambda chains were amplified and inserted into human IgG1 expression vectors. For antibody expression, heavy and light chain expression vectors were transiently transfected into ExpiCHO cells (Thermo Fisher Scientific, A29133) using the ExpiCHO expression system kit. Human IgG1 monoclonal antibody-containing supernatant was harvested and purified by rProtein A Sepharose (GE healthcare), with the resulting monoclonal antibodies collected for further analysis.

To determine the individual gene segments employed by VDJ and VJ rearrangements and the number of nucleotide mutations and amino acid replacements, the variable domain sequences were aligned with germline gene segments using the international ImMunoGeneTics (IMGT) alignment tool (http://www.imgt.org/IMGT_vquest/input)^34^.

### Enzyme-linked immunosorbent assay

The binding of monoclonal antibody to SARS-CoV-2 RBDs, spike, and NTD was evaluated with ELISA. The 96-well microplate (Corning, USA) was coated with SARS-CoV-2 proteins in phosphate-buffered saline (PBS) overnight at 4 °C. After washing, the plate was blocked with 3% (w/v) bovine serum albumin for 2 h at room temperature. After washing, the plate was incubated with monoclonal antibody preparation for 2 h at 37 °C. After washing, the plate was incubated with secondary antibody Rabbit anti-human IgG conjugated to horseradish peroxidase (1:5000 dilution in sterile PBS, Rockland, USA) for 1 h at 37 °C. After washing, the plate was developed by TMB substrate (ThermoFisher, USA) and the reaction was stopped by 1 M sulfuric acid. The absorbance was measured at 450 nm with an ELISA microplate reader. Data were analyzed by Microsoft Excel for Mac version 16.16.27 and graphs were presented by GraphPad Prism version 9.

### ACE2-inhibition assay

The inhibitory activity of monoclonal antibody on the interaction of SARS-CoV-2 RBD and human ACE2 was evaluated using a flow cytometry-based binding assay (Fig. S10a). Serial dilutions of antibodies were mixed with biotinylated RBD and the mixture was incubated with MDCK-ACE2 cells for 1 h at 37 °C. PBS alone was included as the negative control. The mixture of biotinylated RBD with BS-1A (anti-influenza H3 antibody, starting at 100 µg/ml) was included as positive control. After washing, cells were incubated with ExtrAvidin-R-Phycoerythrin conjugate (Sigma-Aldrich, USA) for 30 min at 4 °C. After washing, the binding activities were analyzed by BD FACSCanto II flow cytometer (BD Biosciences, USA). The relative inhibition was calculated as (1- (binding percentage ^antibody^ - binding percentage ^PBS control^) / (binding percentage ^biotinylated RBD^ - binding percentage ^PBS control^)) × 100%. Data were analyzed by Microsoft Excel for Mac version 16.16.27 and graphs were presented by GraphPad Prism version 9.

### RBD-based cross-inhibition assay

Competitive binding assays were performed with monoclonal antibodies as described previously^6^. Briefly, RBD-VLP were coated on 96-well microplates overnight at 4 °C, washed and blocked with dried skimmed milk in PBS for 1 h at room temperature prior to the assays. Antibody was biotinylated using EZ-Link Sulfo-NHS-LC-biotin (Life Technologies, USA) and then mixed with competing antibody (in at least 10-fold excess) and transferred to the blocked plates for 1 h. A second layer Streptavidin-HRP (Life Technologies, USA) was then added and incubated for another 1 h. Plates were then washed, and signal was developed by adding POD substrate (Roche, USA) for 5 min before stopping the reaction with 1 M sulfuric acid. Absorbance (OD450) was measured using a plate reader. The mean and 95% confidence interval of 4 replicate measurements were calculated. The competition was measured as: (X-minimum binding/(maximum binding-minimum binding), where X is the binding of the biotinylated antibody in the presence of a competing antibody. Minimum binding is the self-blocking of the biotinylated antibody or background binding. Maximum binding is the binding of a biotinylated antibody in the presence of non-competing antibody (anti-influenza N1 neuraminidase antibody).

### RBD-based serum competition assay

MDCK-RBD cells were produced by stably transducing MDCK-SIAT1 cells with a Lentiviral vector encoding a cDNA expressing RBD (Wuhan-Hu-1) amino acids 340–538 fused via a short linker to the transmembrane domain of hemagglutinin H7 (A/Hong Kong/125/2017) (EPI977395) at the C-terminal for surface expression^6^. Antibody was biotinylated using EZ-Link Sulfo-NHS-LC-biotin (Life Technologies, USA). The biotinylated antibody of an optimized concentration was mixed with serum dilution and incubated with MDCK-RBD cells for 1 h at 37 °C. After washing, cells were incubated with ExtrAvidin-R-Phycoerythrin conjugate (Sigma-Aldrich, USA) for 30 min at 4 °C. After washing, the binding activities were analyzed by BD FACSCanto II flow cytometer (BD Biosciences, USA) (Fig. S10b). The relative inhibition was calculated as (1- (binding percentage ^serum^ - binding percentage ^PBS control^) / (binding percentage ^biotinylated antibody^ - binding percentage ^PBS control^)) * 100%. Data were analyzed by Microsoft Excel for Mac version 16.16.27 and graphs were presented by GraphPad Prism version 9.

### Pseudotyped SARS-CoV-2 neutralization assay

For production and purification of SARS-CoV-2 pseudotyped lentivirus, the pseudotyped lentivirus carrying SARS-CoV-2 S protein was generated by transiently transfecting HEK-293T (ATCC CRL-3216) cells with pCMV-ΔR8.91, pLAS2w.Fluc.Ppuro and pcDNA3.1-nCoV-S (wild type or variants) using TransITR-LT1 transfection reagent (Mirus). The virus was harvested and clarified at 72 h post-transfection by centrifugation at 4000 *g* for 10 min and 0.45 μm filtering (Pall Corporation) before being aliquoted and stored at −80 °C. The virus titer (transduction units) was determined by AlamarBlue assay according to the manufacturer’s instructions. A total of 1000 transduction units (TU) of SARS-CoV-2 (WT or variants) pseudotyped lentivirus in DMEM (supplemented with 1% FBS and 100 U/ml Penicillin/Streptomycin) were mixed for 1 h at 37 °C with purified and 0.22 μm filtered antibody in 4x serial dilutions from the initial concentration around 0.06 mg/ml (see Source Data file). The mixture was then inoculated with 10,000 HEK293T cells stably expressing human ACE2 in 96-well plates with medium refreshed at 16 h post-infection. Cells were cultured for another 48 h before measuring the relative light units (RLU) in the Bright-Glo Luciferase Assay System (Promega) by Tecan i-control (Infinite 500). The percentage of inhibition was calculated as the ratio of RLU reduction in the presence of each diluted sample to the RLU value of no-sample control and the calculation formula was (RLU ^control^ - RLU ^sample^) / RLU ^control^.

### Sarbecovirus RBD expression and purification

Sarbecovirus RBD constructs p3BNC-RBD-His8-SpyTag003 have previously been described^35^, corresponding to the RBD from SARS-CoV-2 (GenBank ON131086), SARS-CoV (GenBank ON131087), RaTG13-CoV (GenBank ON131088), SHC014-CoV (GenBank ON131089), Rs4081-CoV (GenBank ON131090), pangolin17 (pang17)-CoV (GenBank ON131091), RmYN02-CoV (GenBank ON131092), Rf1-CoV (GenBank ON131093), WIV1-CoV (GenBank ON131094), Yunnan2011 (Yun11)-CoV (GenBank ON131095), BM48-31-CoV (GenBank ON131096) or BtKY72-CoV (GenBank ON131097). Sarbecovirus RBDs were expressed and purified. In brief, expression was carried out in Expi293F cells using the ExpiFectamine 293 Transfection Kit (both Thermo Fisher) according to the manufacturer’s instructions, and cell suspensions were harvested 5 days after transfection. Supernatants were supplemented with cOmplete Mini EDTA-free Protease Inhibitor Cocktail (Roche), clarified by centrifugation at 4,000 g and 4 °C for 5 min, then passed through a 0.45 μm syringe filter (Thermo Fisher). Purification was performed by SpySwitch^15^ (coupled to SulfoLink Coupling Resin) in batch format at 4 °C. Supernatants were supplemented with 10% (v/v) 500 mM Tris-HCl pH 7.5 + 3 M NaCl, incubated with SpySwitch resin for 1 h, washed with 4× 10 column volumes (CV) 50 mM Tris-HCl pH 7.5 + 300 mM NaCl. Proteins were eluted six times with 1.5 CV of 50 mM acetic acid pH 5.0 + 150 mM NaCl, incubating each fraction for 5 min. Each elution fraction was neutralized by collection in a microcentrifuge tube containing 0.3 CV 1 M Tris-HCl pH 8.0. Protein concentrations were determined by bicinchoninic acid assay (BCA Protein Assay Kit, Thermo Fisher).

### Enzyme-linked immunosorbent assay (sarbecovirus RBD)

The binding of mAb to various sarbecovirus RBDs was assessed using ELISA. Nunc MaxiSorp flat-bottom plates (Thermo Fisher) were coated with PBS containing 50 nM of specified RBD by incubating for 16 h at 4 °C. The plate was incubated for 2 h at 25 °C with Blocking Buffer: 5% (w/v) Skim Milk in PBS pH 7.4. The plates were washed three times with PBS pH 7.4 with 0.1% (v/v) Tween 20 (PBST). The plate was then incubated for 1 h with 50 nM of specified antibody diluted in Blocking Buffer and then washed three times with PBST. The plate was incubated at 25 °C for 1 h with 1/2500 dilution of goat anti-human IgG HRP antibody (Sigma-Aldrich A8667) in Blocking Buffer and then washed three times with PBST. The plate was finally incubated for 2 min with TMB substrate (Thermo Scientific), before the reaction was stopped with 1 M sulfuric acid. The absorbance was measured at 450 nm with a FLUOstar Omega microplate plate reader (BMG Labtech). The mean absorbance from triplicates is presented at a heat map. The results shown are representative of two separate experiments.

### Pseudotyped sarbecovirus neutralization assay

SARS-CoV, WIV1, and SHC014 pseudoviruses based on HIV-1 lentiviral particles were prepared as described^17,21,35,52^ using genes encoding S protein sequences with cytoplasmic tail deletions: 21 amino acid deletions for WIV1 and SHC014, and a 19 amino acid deletion for SARS-CoV. Plasmids expressing the spike protein found in the bat (*Rhinolophus sinicus*) coronavirus bCoV-WIV16 as well as the Sunda pangolin (*Manis javanica*) coronaviruses from Guandong, China (pCoV-GD) and Guanxi, China (pCoV-GX) have been described previously and are based on ALK02457 (GenBank), Pangolin_CoV_EPI_ISL_410721 (Gisaid) and Pangolin_CoV_EPI_ISL_410542 (Gisaid)^53^. For neutralization data presented in Fig. 2b, four-fold dilutions of purified IgGs (starting concentrations of 75 µg/ml) were incubated with a pseudotyped virus for 1 h at 37 °C. The pseudotyped virus and IgG mixture was added to 293TACE2 target cells and incubated at 37 °C for 48 h. Cells were lysed with Luciferase Cell Culture Lysis 5x reagent (Promega) and luciferase activity in lysates was measured using the Nano-Glo Luciferase Assay System (Promega). Relative luminescence units (RLUs) were normalized to values derived from cells infected with pseudotyped virus in the absence of IgG. Half-maximal inhibitory concentrations (IC_50_ values) were determined using 4- or 5-parameter nonlinear regression in AntibodyDatabase^54^.

### Crystallization, data collection and structure determination

The RBD domain (333-530) of SARS-CoV-2 WT Spike was constructed and cloned into the pTT vector using oligonucleotide primers (gtggggtaccacaaacctgtgcccatttg and gtggggatccgtggtggtgatggtgatgggacttcttggggccgcac) for protein expression in HEK293 EBNA (ATCC CRL-10852) suspension cells by transient transfection using polyethyleneimine (PEI) followed by 37 °C incubation for 4 days. Culture supernatants were harvested and clarified by centrifugation at 6500 g for 20 min, followed by Ni-NTA affinity (GE Healthcare) purification and Superdex 200 Increase 10/300 GL (GE Healthcare) chromatography in a buffer containing 20 mM Tris/HCl, pH 7.5, 150 mM NaCl. Fab fragments were digested from IgG using the immobilized papain resin (Thermo Scientific) for 24 h at 37 °C and purified with rProtein A Sepharose (GE Healthcare). Purified RBD and Fab were mixed at 1:1.15 molar ratio for 30 min incubation on ice before further purification by Superdex 200 Increase 10/300 GL (GE healthcare) in 20 mM Tris/HCl, pH 8.0, 150 mM NaCl. The complex peak was verified by 14% SDS-PAGE, pooled together and concentrated to higher than 12 mg/ml for hanging-drop vapor diffusion crystallization at 20 °C. For FP-12A, crystals appeared on day 2 in the condition containing 0.2 M Ammonium sulfate; 0.05 M Magnesium sulfate heptahydrate; 0.1 M Bicine pH 9.0; 20 % v/v PEG Smear Medium. For IS-9A, crystals appeared on day 2 in the condition containing 2% (v/v) 1,4-Dioxane, 0.1 M Tris pH 8.0 and 15% (w/v) Polyethylene glycol 3,350. For IY-2A, crystals appeared on day 6 in the condition containing 0.2 M Ammonium citrate tribasic pH 7.0, 0.1 M Imidazole pH 7.0, and 20% (w/v) Polyethylene glycol monomethyl ether. Crystals were harvested with diffraction datasets collected at National Synchrotron Radiation Research Center (NSRRC) BL15A1 or TPS05A beamline, at a temperature of 100 K with the wavelength 1.00 (Table S2). Data were processed in iMosflm 7.2.2^55^. Molecular replacement was performed by Phenix 1.19.2-4158 Phase-MR^56^ using structures of RBD and Fab as separated searching ensembles. The solved structure was further refined in phenix.refine with manual adjustments done in WinCoot 0.8.9^57^. Structural figures were prepared with UCSF-ChimeraX 1.3^58^ and PyMOL 2.5^59^.

### Cryo-EM sample preparation, data collection, processing, and model building

SARS-CoV-2 (Delta or Omicron BA.1) Spike protein sequences (14-1209, S2P) were synthesized (service provided by Genomics, Taiwan) and cloned into the pTT vector by restriction digestion and ligation (KpnI and EcoRV) for expression in HEK293 EBNA (ATCC CRL-10852) suspension cells by transient transfection using polyethyleneimine (PEI) followed by 32 °C incubation for 6 days. Culture supernatants were harvested and clarified by centrifugation at 6,500 g for 20 min, followed by Ni-NTA affinity (GE Healthcare) purification and Superose 6 Increase 10/300 GL (GE Healthcare) gel filtration in a buffer containing 20 mM Tris/HCl, pH 7.5, 150 mM NaCl. Antibody expression and Fab purification were the same as mentioned above. The ratio of Spike and Fab for complex formation as well as the incubation time was evaluated by negative stain transmission electron microscopy. Freshly purified Spike (diluted to 1 mg/ml) was mixed with Fab (2 mg/ml) at 3:1 (v/v) ratio in 20 mM Tris pH 8.0, 150 mM NaCl for 3 min at room temperature before being applied (3 µl) to a glow discharged Quantifoil R1.2/1.3 holey carbon grid mounted in a Mark IV Vitrobot (Thermo Fisher Scientific) at 4 °C with 100 % humidity. Grids were blotted at force 0 for 3 seconds. Data were collected on a Titan Krios G3 at 300 kV (Thermo Fisher Scientific), equipped with a Gatan K3 detector and the Gif Quantum energy filter with 20 eV slit width. Movies were acquired in EPU (Thermo Fisher Scientific, v2.10) at two exposures per hole. Total electron dose was 38 e^-^ /Å^2^ collected over 2.5 s and fractionated into 40 frames. The corresponding pixel size was 0.83 Å (for IS-9A) and 1.06 Å (for IY-2A) in the defocus range from −1.5 to −2 µm. Data were processed with C1 symmetry in CryoSPARC 3.0 (for IS-9A)^60^ or relion 3.0 (for FP-12A and IY-2A)^61^ with the resolution of the final volume determined by gold-standard Fourier shell correlation (FSC) cutoff at 0.143 (Fig. S11). The coordinates used for model fit in map included PDB 7CAK and 6ZDH, as well as crystal structures solved in this study.

### Binding affinity determination using biolayer interferometry

The spike protein (SARS-CoV-2 Delta S2P or Omicron S6P) was conjugated with biotin using Biotinylation Kit (Sigma-Aldrich) according to manufacturer’s instructions. Biotinylated spike was loaded at 50 μg/ml in PBS onto streptavidin biosensors (Molecular Devices, ForteBio). Association and dissociation of IgG by biotinylated spike was performed in PBS throughout all the steps at indicated concentrations (Fig. S1) for 5 min and 10 min, respectively. The kinetic and equilibrium constants were not determined since there could be avidity effects in the experiment (bivalent IgG interacting with trimeric spike).

### Reporting summary

Further information on research design is available in the Nature Portfolio Reporting Summary linked to this article.

## Supplementary information

Supplementary Information

Reporting Summary

## Data Availability

The data associated with this study are available within the article, its supplementary information and Source Data file. Source data are provided with this paper. The coordinates and structure factors of the SARS-CoV-2 RBD/FP-12A, RBD/IS-9A and RBD/IY-2A crystallographic complexes generated in this study have been deposited in the PDB (Protein Data Bank) under accession codes 8HHF, 8HHG and 8HHH, respectively. Cryo-EM volumes and structure models of the SARS-CoV-2 Delta Spike/FP-12A, Delta Spike/IS-9A and BA.1 Spike/IY-2A generated in this study have been deposited in the EMDB (Electron Microscopy Data Bank) under accession codes EMD 34806, EMD 34807, and EMD 34808, and in the PDB under accession codes 8HHX, 8HHY and 8HHZ, respectively. Source data are provided with this paper.

## Source data

Source Data
