# Supplementary material for: Structural basis for a conserved neutralization epitope on the receptor-binding domain of SARS-CoV-2
Source: Nat Commun. 2023 Jan 19;14:311. doi: 10.1038/s41467-023-35949-8 (PMC9852238; doi:10.1038/s41467-023-35949-8)
Supplement: Supplementary file 1 — Supplementary Information [file 41467_2023_35949_MOESM1_ESM.pdf]

Table S1. Anti-SARS-CoV-2 RBD monoclonal antibody heavy and light chain variable domain gene usage.

mAb	H-L	Vh	Jh	Dh	rf	V_h junction sequence	nt Mut	aa Sub	Vl	Jl	V_l Junction Sequence	nt Mut	aa Sub
EY-6A	H-K	3-30*03 or 18 or 3-30-5*01 F	4*02 F	2-21*01 F	1	CAKDGGKLWVYYFDYW	6	5	1-39*01 F or 1D-39*01 F	4*01 F	CQQSYSTLALTF	0	0
FP-12A	H-λ	3-30*03 or 18 or 3-30-5*01 F	6*02 F	3-10*01 F	1	CANGFGEYYYYAMDVW	1	1	6-57*02 F	3*02 F	CQSYDSSNWVF	3	2
IV-6D	H-K	3-9*01 F	4*02 F	5-18*01 F	1	CAKGHTYTAMLRMAFDYW	7	4	3-15*01 F	5*01 F	CQQYNNWPPSITF	0	0
IV-4B	H-λ	3-9*01 F	4*02 F	3-10*01 F	1	CAKGRLGELLFPVDYW	6	5	1-44*01 F	3*02 F	CAAWDDSLNGWVF	2	1
IV-10C	H-λ	4-39*07 F	4*02 F	3-10*01 F	1	CATLLWLRGYFDYW	6	6	6-57*01 F	3*02 F	CQSYDSSNWVF	0	0
IS-9A	H-K	5-10-1*03 F	4*02 F	3-22*01 F	2	CARGRNYLDSRGRFDYW	9	6	1-33 or 1D-33*01 F	3*01 F	CQQYDSLGFTF	2	2
IS-11B	H-K	5-10-1*03 F	4*02 F	3-22*01 F	2	CARGRSYYDSRGRFDYW	3	3	1-33*01 F or 1D-33*01 F	3*01 F	CQQYDSLGFTF	3	3
IY-2A	H-λ	4-34*01 F	3*01 F	3-3*02 F	3	CARGLGIFGVVTLSDVW	9	4	6-57*02 F	3*02 F	CQSYDSGIWVF	3	2

Abbreviations: H, heavy; K, kappa; **λ**, lambda; Vh, variable gene segment of the heavy chain variable domain; Dh, diversity gene segment of the heavy chain variable domain; Jh, joining gene segment of the heavy chain variable domain; Mut, number of nucleotide mutations; Sub, number of amino acid substitutions; Vl, variable gene segment of the light chain variable domain; Jl, joining gene segment of the light chain variable domain; rf, reading frame.

Table S2. X-ray data collection and refinement statistics.

	RBD/FP-12A	RBD/IS-9A	RBD/IY-2A
**Data collection**			
Wavelength	0.9998	1.0000	1.0000
Space group	*P* 1	*P* 1 2_1 1	*C* 1 2 1
Cell dimensions			
a, *b*, *c* (Å)	58.83, 79.12, 92.54	85.28, 86.86, 98.09	193.02, 225.15, 95.52
α, β, γ (°)	76.26, 81.16, 71.13	90, 108.66, 90	90, 96.16, 90
Resolution (Å)	32.25 - 2.6 (2.693 - 2.6)	32.62 - 2.13 (2.206 - 2.13)	33.64 - 2.85 (2.952 - 2.85)
R_{sym} or R_{merge}	0.080 (0.753)	0.043 (0.211)	0.080 (0.416)
$I / \sigma I$	5.6 (1.4)	9.8 (3.6)	6.6 (1.8)
Completeness (%)	97.8 (97.1)	99.66 (97.63)	99.88 (99.99)
Redundancy	4.0 (4.0)	3.7 (3.0)	3.8 (3.8)
**Refinement**			
Resolution (Å)	28.22 - 2.49 (2.58 - 2.49)	28.22 - 2.49 (2.58 - 2.49)	33.64 - 2.85 (2.952 - 2.85)
No. reflections	45959	75685	94123
R_{work} / R_{free}	0.230 / 0.264	0.176 / 0.217	0.211 / 0.250
No. atoms	8315	10907	19091
Protein	8101	9717	19063
Ligand/ion	87	38	28
Water	127	1152	0
B-factors ($Å^2$)	72	27	64
Protein (RBD)	47	30	66
Protein (Fab)	88	26	64
Ligand/ion	96	67	79
Water	51	33	-
R.m.s. deviations			
Bond lengths (Å)	0.004	0.008	0.010
Bond angles (°)	0.66	0.99	1.18
Rotamer outliers (%)	3.02	2.40	4.44
Clash score	9.09	5.81	6.27
Ramachandran			
Favored (%)	91.48	97.09	94.24
Allowed (%)	8.03	2.91	5.68
Outliers (%)	0.48	0.00	0.08
Wilson B-factor ($Å^2$)	53	23	55

One crystal was used for each dataset in structural determination.

Table S3. Cryo-EM data collection, refinement and validation statistics.

	Spike-FP-12A (Delta variant) EMDB 34806 PDB 8HHX	Spike-IS-9A (Delta variant) EMDB 34807 PDB 8HHY	Spike-IY-2A (BA.1 variant) EMDB 34808 PDB 8HHZ
**Data collection and processing**			
Magnification	105,000	105,000	81,000
Voltage (kV)	300	300	300
Electron exposure ($e^{-}/Å^2$)	54.06	54.06	54.06
Defocus range (μm)	-1.5 ~ -2.5	-1.3 ~ -2.2	-1.5 ~ -2.5
Pixel size (Å)	0.83	0.83	1.06
Symmetry imposed	C1	C1	C1
Initial particle images (no.)	1,133,890	1,542,976	1,405,490
Final particle images (no.)	120,515	296,356	67,276
Map resolution (Å)	3.62	2.77	4.28
FSC threshold	0.143	0.143	0.143
**Refinement**			
Initial model used (PDB code)	7CAK	7K43	7XOD
Model resolution (Å)	3.6	2.8	4.2
FSC threshold	0.143	0.143	0.143
Map sharpening *B* factor ($Å^2$)	-150	-112.7	-150
Model composition			
Non-hydrogen atoms	27451	27423	32301
Protein residues	3477	3483	4286
Ligands	36	32	0
B factors ($Å^2$)			
Protein	241	109	114
Ligand	269	106	-
R.m.s. deviations			
Bond lengths (Å)	0.003	0.004	0.003
Bond angles (°)	0.697	0.674	0.646
Validation			
MolProbity score	2.00	2.06	2.13
Clashscore	12.82	14.77	18.01
Poor rotamers (%)	0.10	0.00	0.00
Ramachandran plot			
Favored (%)	94.30	94.28	94.48
Allowed (%)	5.62	5.64	5.49
Disallowed (%)	0.00	0.09	0.02

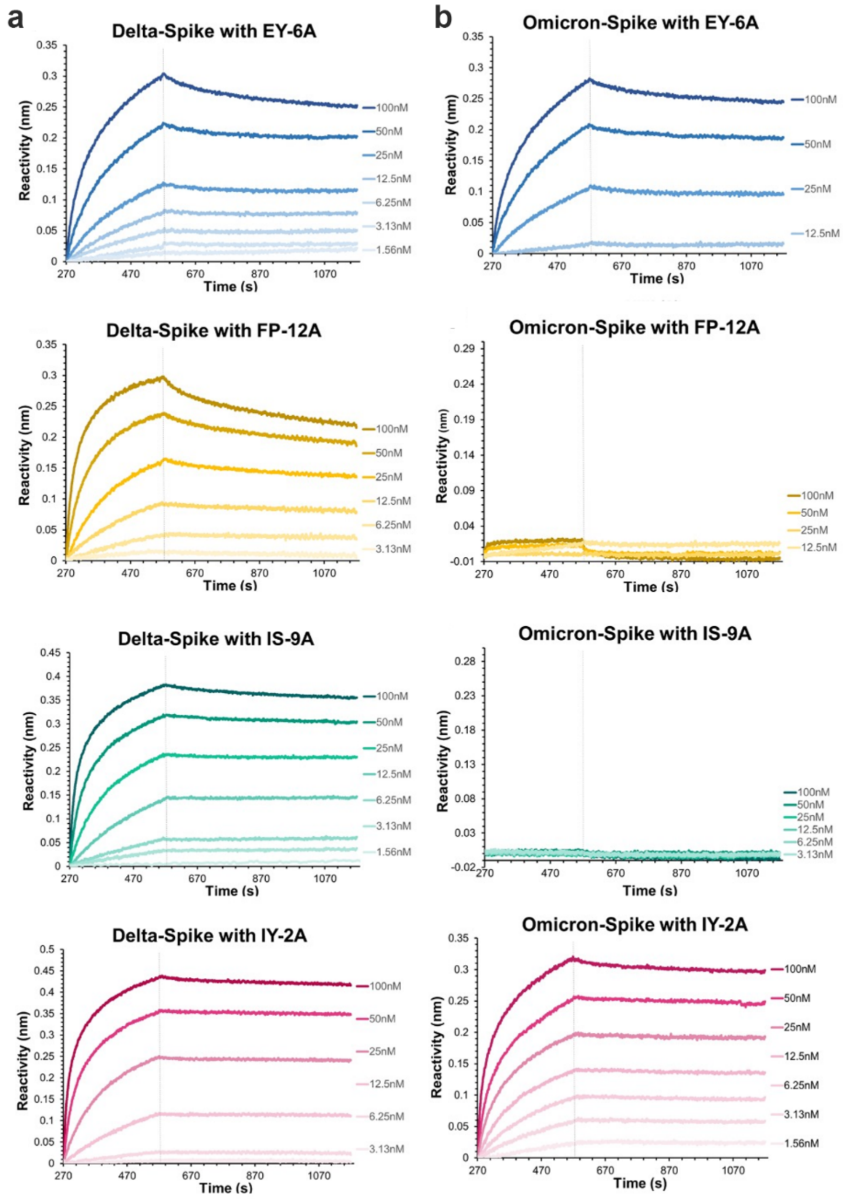

Figure S1. Binding activities of class 4 antibodies with Delta and Omicron Spike. (a) The binding curve of EY-6A (blue), FP-12A (yellow), IS-9A (teal) and IY-2A (red) with Delta Spike. **(b)** The binding curve of EY-6A (blue), FP-12A (yellow), IS-9A (teal) and IY-2A (red) with Omicron BA.1 Spike. The x-axis shows reaction time and the y-axis the reading of bio-layer interferometry. FP-12A and IS-9A have completely lost affinity with Omicron BA.1 Spike. The kinetic and equilibrium constants were not determined since there could be avidity effects in the experiment (bivalent IgG interacting with trimeric spike).

a

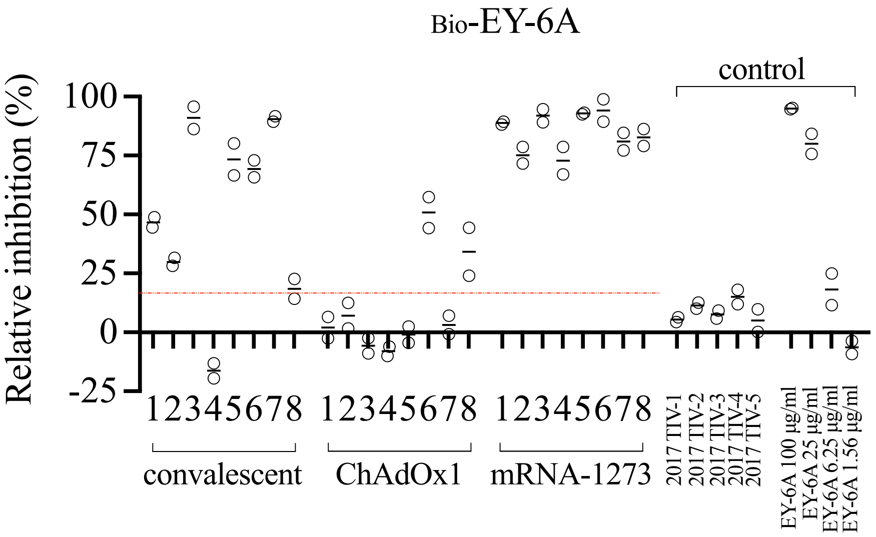

b

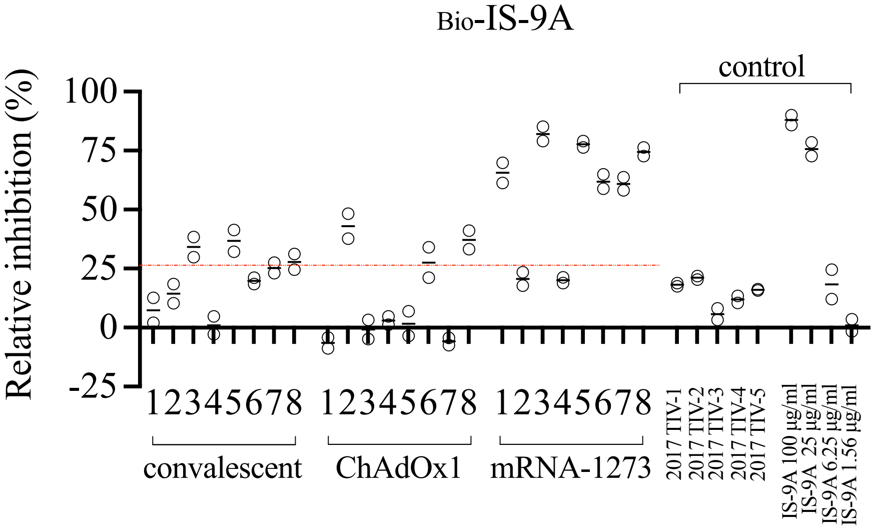

c

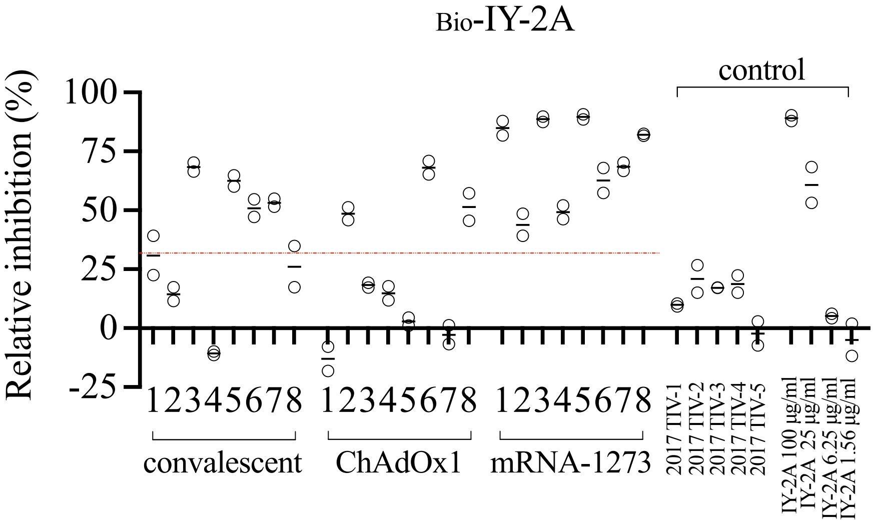

Figure S2. Class 4 antibodies compete with serum for RBD binding. Competition binding results of (**a**) EY-6A, (**b**) IS-9A, and (**c**) IY-2A with convalescent and post-vaccination sera

using MDCK-RBD cells. All sera were diluted 1:10 for analysis and each sample was run with two technical replicates (n=2). The black line represents the mean. The cut-off was defined by mean plus two standard deviation of control sera (after trivalent influenza vaccine) collected in 2017 and represented by the red dotted line. Self-competition using purified mAb was included in the experiment. Anti-influenza H3 mAb BS-1A was included as the negative control. TIV; trivalent influenza vaccine. Source data are provided as a Source Data file.

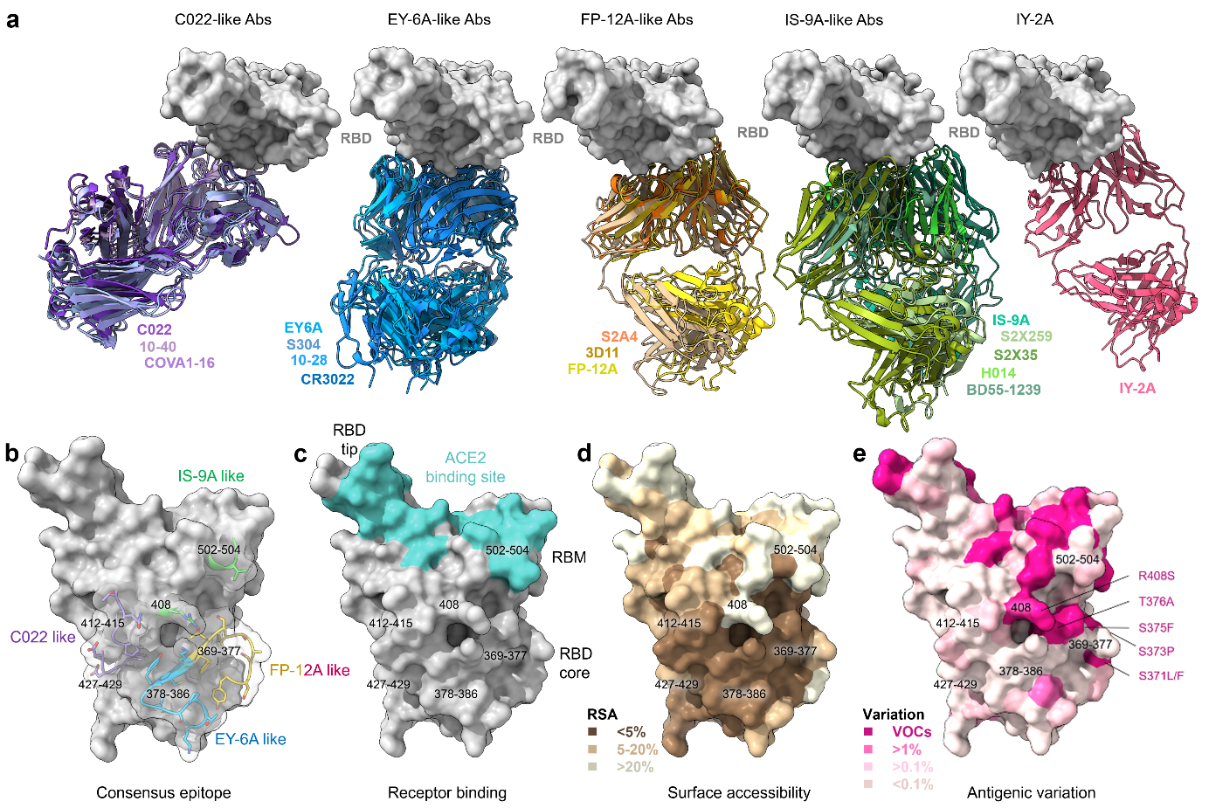

Figure S3. The binding mode of class 4 antibodies and the structural properties of RBD. (a) Superimposition of the Fab from all known class 4 antibodies (ribbons, colored by subgroups and specified by labels) bound to RBD (surface, light gray). C022 and similar antibodies: purple; EY-6A and similar antibodies: blue; FP-12A and similar antibodies: yellow; IS-9A and similar antibodies: green; IY-2A: red. PDB code 7RKU for C022, 7SD5 for 10-40 and 7JMW for COVA1-16. PDB code 6ZER for EY-6A, 6W41 for CR3022, 7R6X for S304 and 7SI2 for 10-28. PDB code 7M7B for 3D11 and 7JVA for S2A4. PDB code 7CAH for H014, 7R6W for S2X35, 7M7W for S2X259, and 7WRL for BD55-1239. **(b)** Mapping the consensus epitope for each group of the class 4 antibodies, drawn as ribbon and sticks, labeled with residue range and colored accordingly as in (a). **(c)** Mapping of the receptor binding motif (RBM, cyan) on RBD (gray). **(d)** Mapping of the relative surface accessibility (shown as a brown heat map) on RBD. Relative surface accessibility (RSA) was calculated from the up conformation of RBD based on a previous report[33]. **(e)** Mapping of the sequence variation (shown as a magenta heat map) on RBD. The darkest color highlights residues of all the known Variants of Concern (VOCs), and those that overlap with the class 4 antibody epitope are labeled on the right.

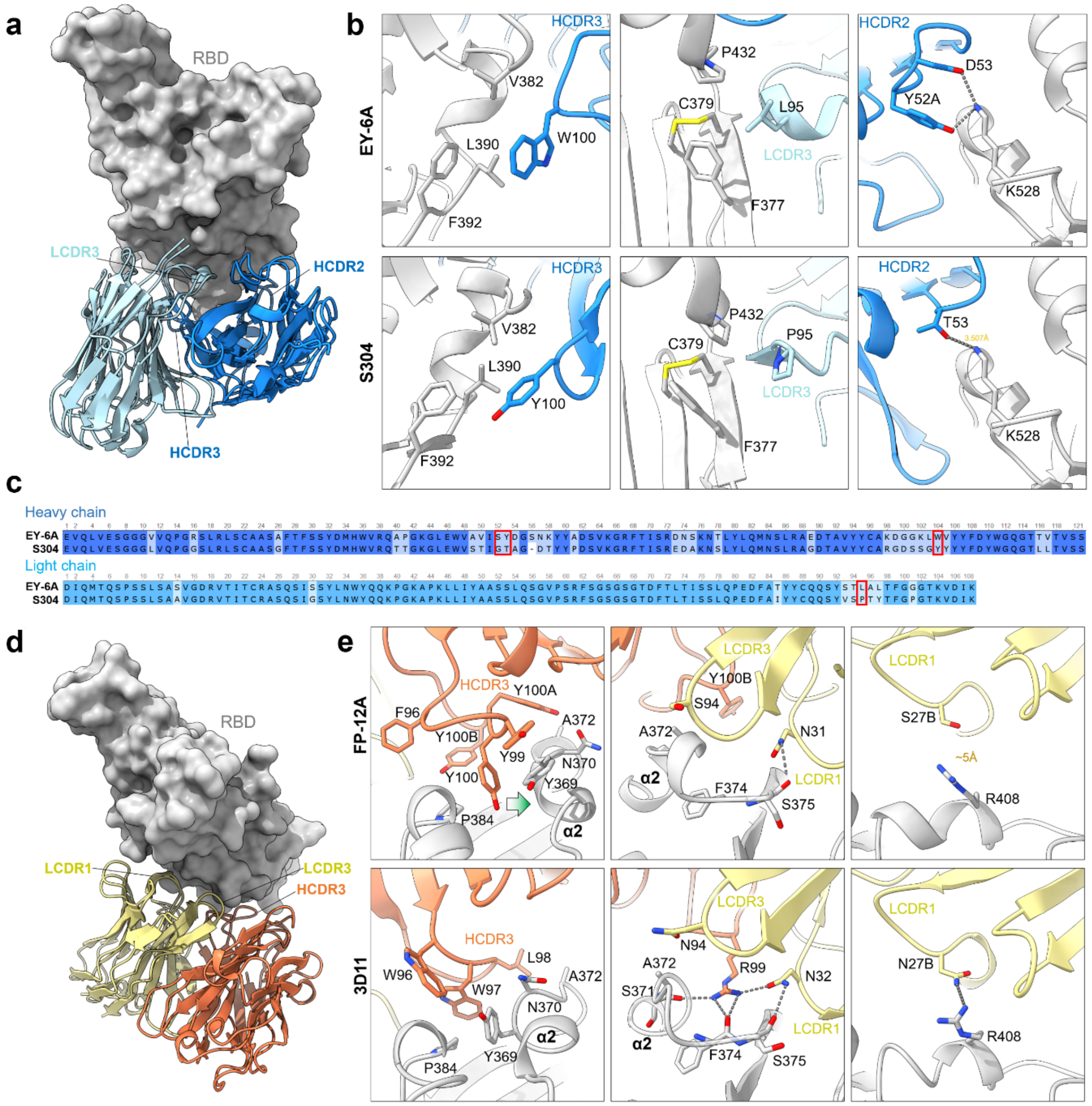

Figure S4. Structural comparison between mAbs EY-6A and S304 and between mAbs FP-12A and 3D11. (a) Superimposition of the overall structure of EY-6A-bound and S304-bound RBD (PDB code 6ZER for EY-6A and 7R6X for S304). RBD is shown in gray surface. Both Fabs are shown in ribbons in the same color, with heavy chains colored in dark blue and light chains light blue. CDRs that are shown in more details in (b) are labeled. **(b)** Selected views of the detailed structural difference between EY-6A and S304 in HCDR3, LCDR3 and HCDR2 as labeled. Key interacting residues are drawn in sticks and labeled. Hydrogen bonds are drawn as gray dotted lines. **(c)** Sequence alignment of EY-6A and S304 with the indicated residues (as in B) highlighted in red boxes. **(d)** Superimposition of the overall structure of FP-12A-bound and 3D11-bound RBD (PDB code 7M7B for S304). RBD is shown in gray surface. Both Fabs are shown in ribbons in the same color, with heavy

chains colored in orange and light chains yellow. CDRs that are shown in more details in (B) are labeled. **(e)** Selected views of the detailed structural difference between FP-12A and 3D11 in HCDR3, LCDR3 and LCDR1 as labeled. Key interacting residues are drawn in sticks and labeled. A small shift of the 365-371 helix induced by FP-12A HCDR3 is indicated by a green arrow.

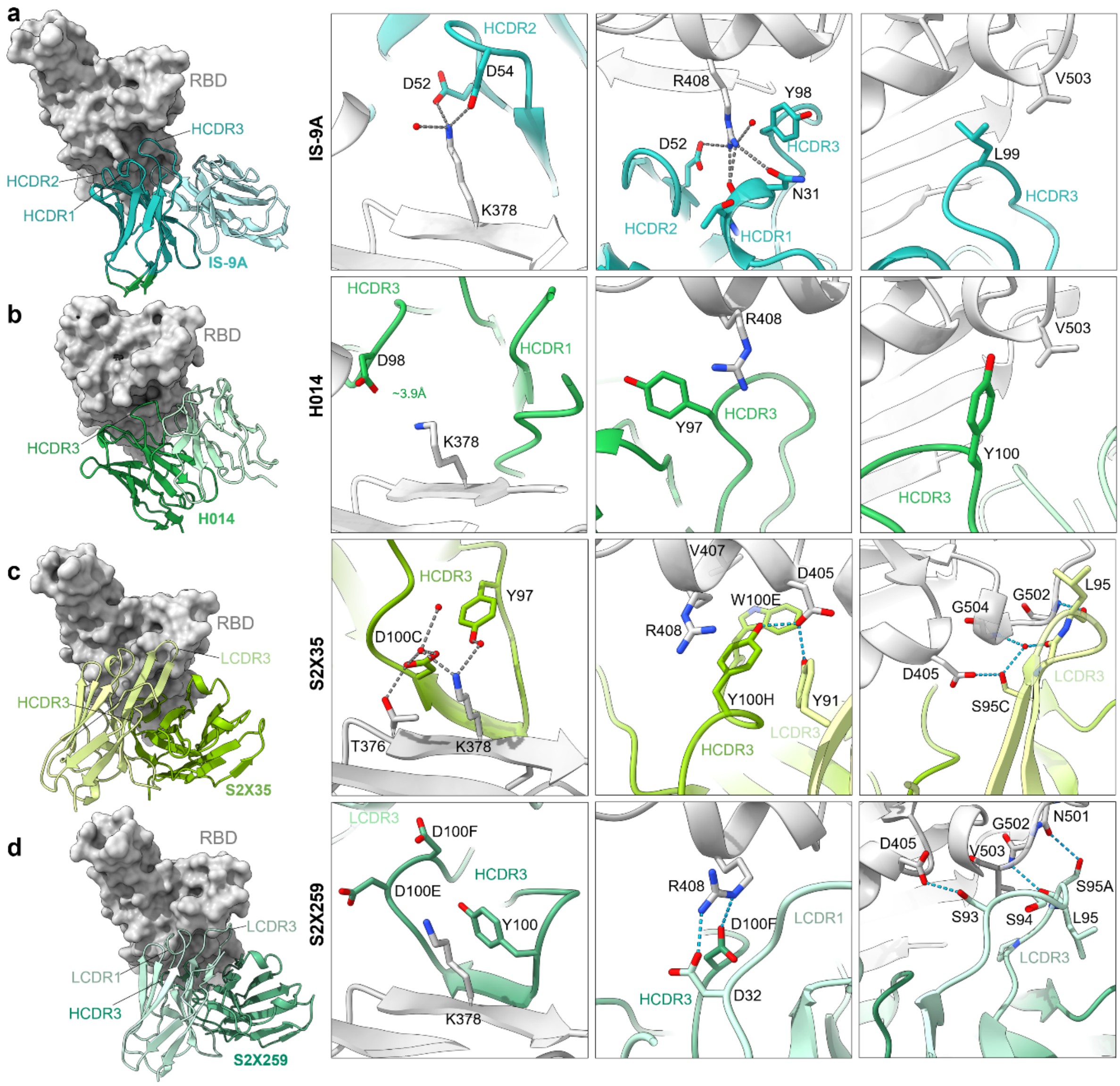

Figure S5. Structural comparison between IS-9A and other similar mAbs. (a) The overall structure of IS-9A-bound RBD. RBD is shown in gray surface, and Fab is shown in ribbons with heavy and light chains colored in dark and light teal. CDRs that are shown in more details are labeled. Selected views of the detailed interaction with key residues of RBD (K378, R408 and the 502-504 region) are shown on the right panels for comparison. **(b)** The same structural presentation of the H014-bound RBD is shown as in (a). Heavy and light chains are colored in dark and light green. PDB code 7CAH. **(c)** The S2X35-bound RBD is shown as in (a). Heavy and light chains are colored in dark and light yellowgreen. PDB code 7R6W. **(d)** The S2X259-bound RBD is shown as in (a). Heavy and light chains are colored in dark and light seagreen. PDB code 7M7W. Colors are consistent with Fig. 3c. The interface of C118 or C022 with RBD is not shown for comparison due to a significantly different binding mode.

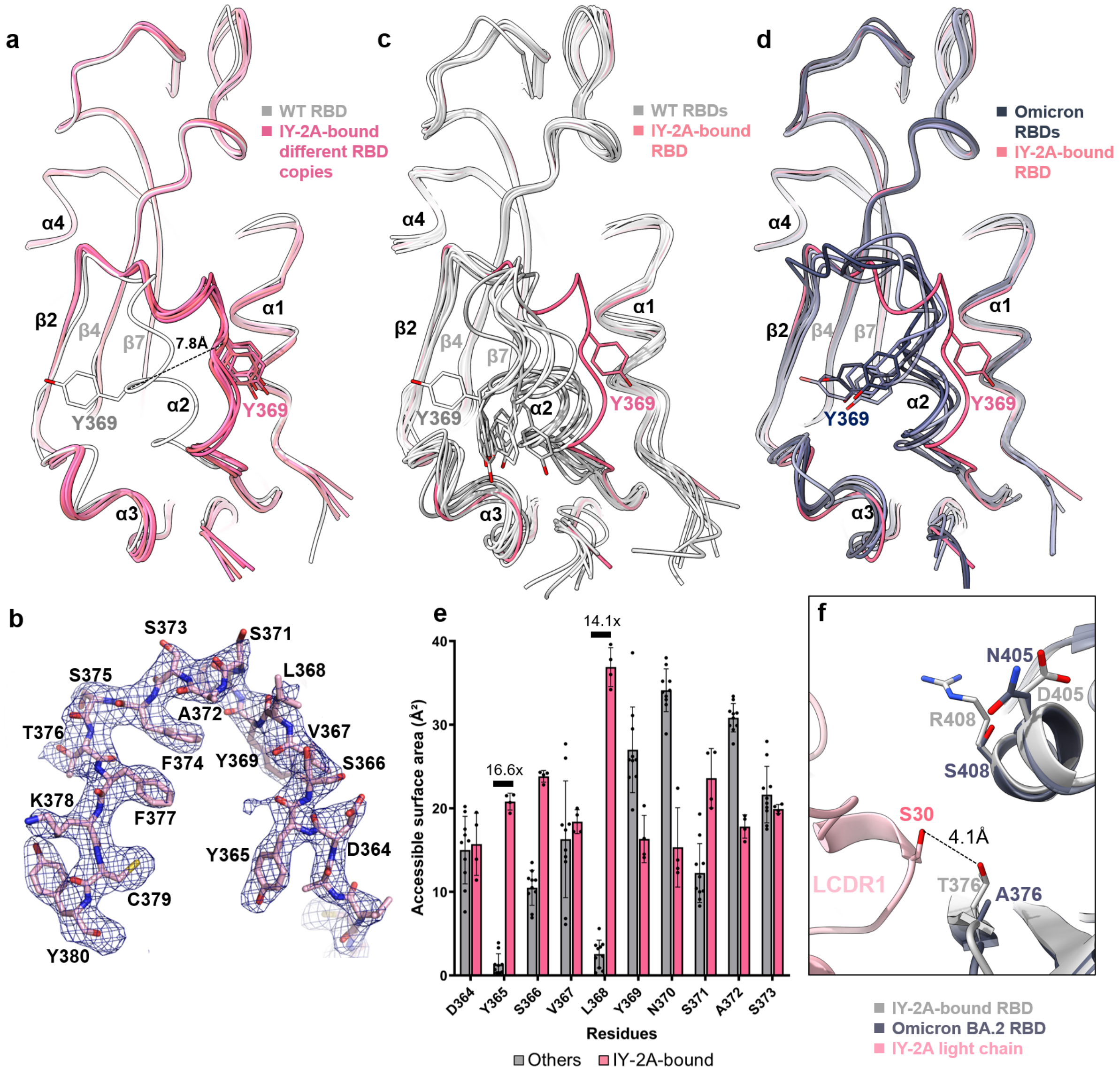

Figure S6. Structural flexibility and surface accessibility of the 364-376 region of RBD in the IY-2A-bound structure. (**a**) Superimposition of four copies of the IY-2A-bound RBD (different pink colors) in one asymmetric unit shows a consistent conformation of this region. Y369 is highlighted as sticks, showing the displacement of the Cα atom for 7.8Å from the ACE2-bound WT RBD (PDB 6M0J). (**b**) The electron density of the difference map surrounding the 364-373 region of RBD. σ level at 3.0. (**c**) Superimposition of ten WT RBD (PDB code: 6M0J, 6ZER, 7M7W, 7R6X, 7R6W, 6W41, 7RKU and 7JMW, the IS-9A-bound and the FP-12A-bound RBD structures, in light gray shades) shows a conserved conformation of the 364-376 region (α2 helix and α2-β2 linker) in comparison with the IY-2A-bound RBD (pink), with Y369 highlighted in sticks. (**d**) Superimposition of the WT RBD (PDB 6M0J, gray), five Omicron RBDs (BA.1: PDB 7XAZ, 7XO6; BA.2: PDB 7XB0, 7ZF7, 7XOC, different purple shades) and the IY-2A-bound RBD (pink) shows structural

variation of this region. (**e**) A bar chart of the solvent accessible surface area (ASA) calculated for residues 364-373 of ten other RBD structures (the same as panel a left, in gray) compared to the IY-2A-bound RBD (four copies as panel b, in pink). Two residues (365 and 368) with more than 10-time increase of ASA are indicated with the fold change above the bar. Data are mean ± standard deviation with n=4 for IY-2A structure and n=10 for others. (**f**) The enlarged view of the IY-2A-bound RBD (gray) superimposed with the Omicron BA.2 RBD (PDB 7ZF7, dark purple) shows a relatively close distance between S30 of LCDR1 (pink) and T376 of RBD (4.1Å). Residues at 405 or 408 of RBD are not involved in any interaction.

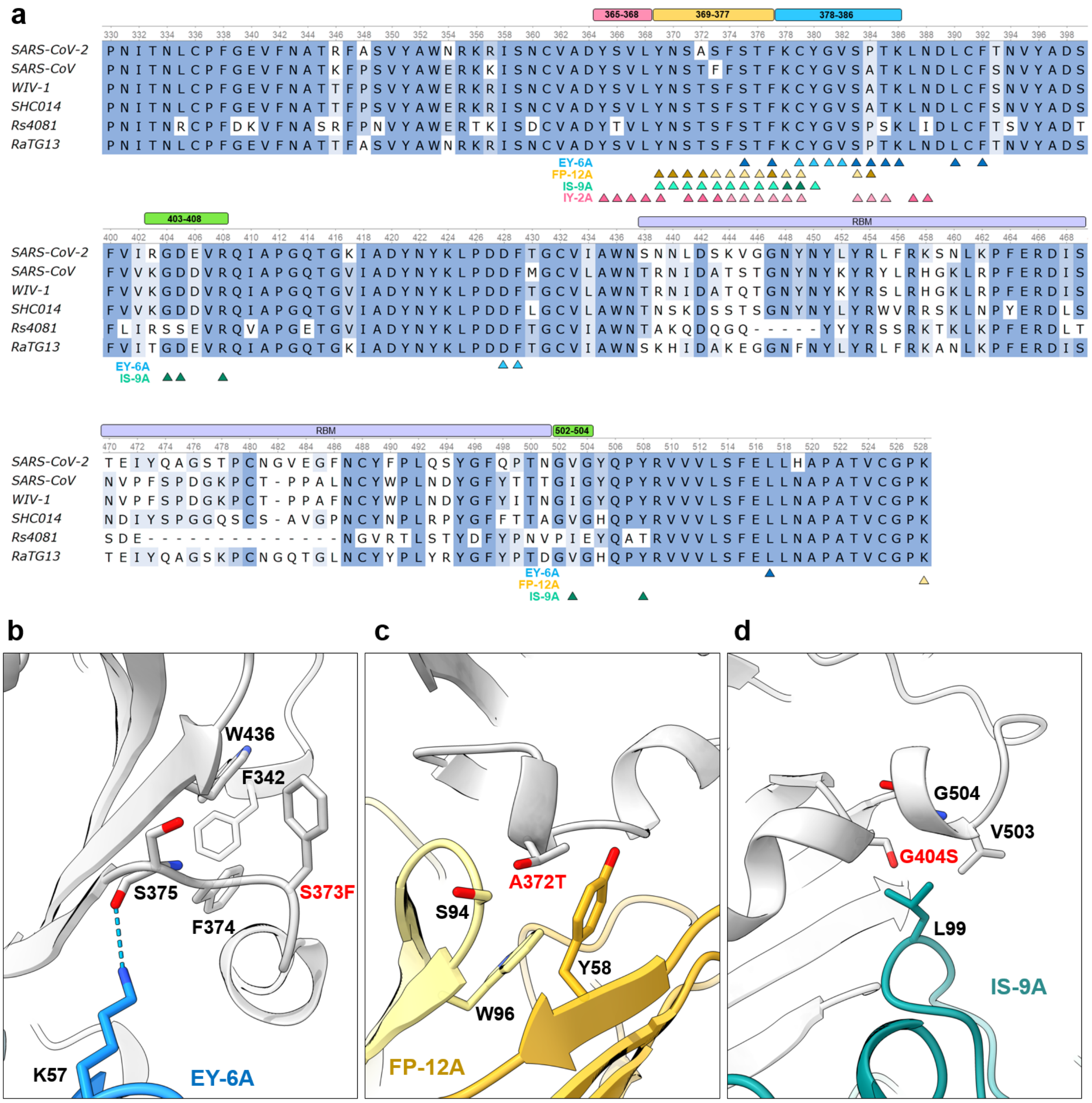

Figure S7. Sequence alignments of RBD from various sarbecovirus strains and footprint mapping. (a) Sequences are aligned following the residue numbering of SARS-CoV-2 spike. Overlaying shades indicate the conservation of each residue: the darker, the more conserved. The RBM and featured epitope regions of class 4 antibodies are shown as bars above the sequence colored accordingly as Fig. S2B-C. Recognition residues of each antibody (EY-6A: blue; FP-12A: yellow; IS-9A: teal; IY-2A: red) are shown below the sequence as triangles, with the darker color by heavy chain and lighter color by light chain. **(b)** Modelling of S375F mutation (as in SARS-CoV) on RBD implicates a potential different packing with surrounding aromatic side chains, which may alter the position of S375 and its hydrogen bonding with K57 of EY-6A. The dotted line shows the hydrogen bond. (**c**) Modelling of A372T mutation (as in all other sarbercoviruses than SARS-CoV-2) shows an insufficient

space for the side chain of Thr when interacting with FP-12A. (**d**) Modelling of G404S mutation (as in Rs4081) reveals an unfavorable contact with the hydrophobic residue L99 of IS-9A, while the local structure may also change due to the closely packed residues nearby.

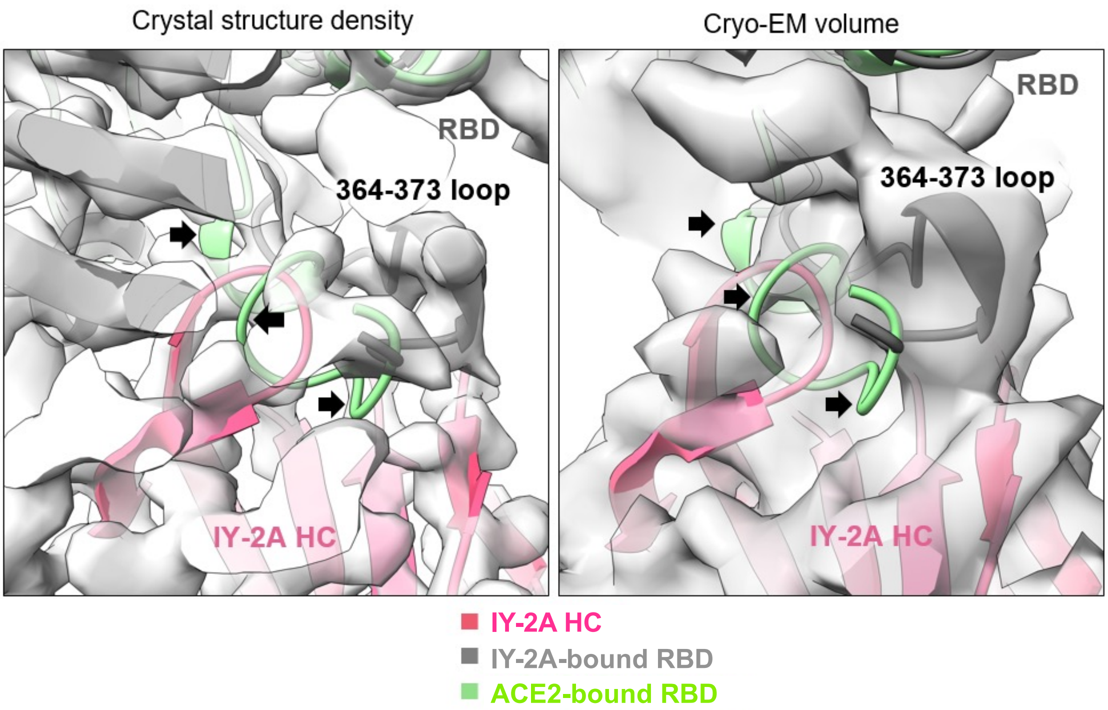

Figure S8. Comparison of electron density in crystal structure (left) and cryo-EM volume (right) in the region where the 364-375 loop of RBD undergoes conformational change. IY-2A heavy chain is colored in pink. The IY-2A-bound RBD in grey and ACE2-bound RBD (PDB code 6M0J) in light green, with black arrows highlighting the out-of-density regions. For a clearer view of electron density in the crystal structure, please refer to Fig. S6b.

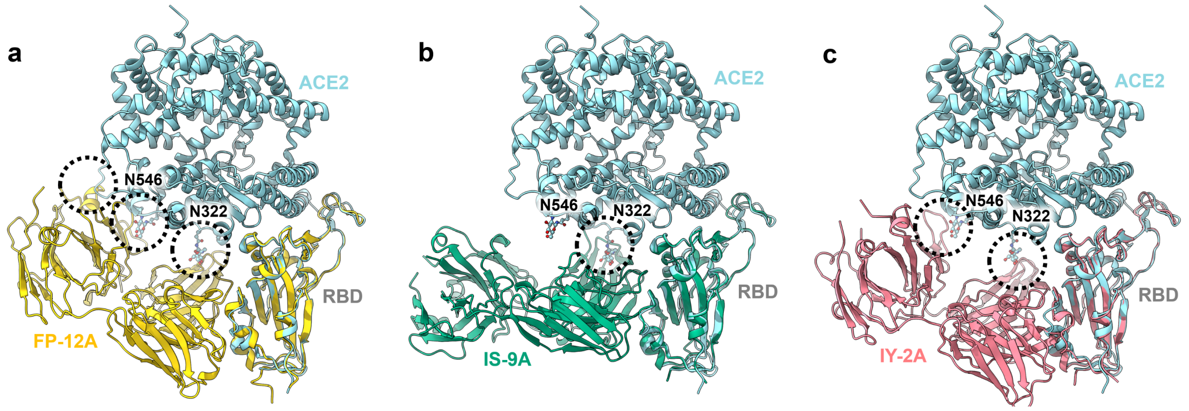

Figure S9. Steric clashes with ACE2 of the class 4 antibodies. The superimposition of Fab-RBD complexes with the ACE2-bound RBD shows clashes (black dashed circles) between FP-12A (yellow) **(a)**, IS-9A (green) **(b)** or IY-2A (pink) **(c)** with ACE2 (cyan), especially in two glycosylation sites, N322 and N546. The first GlcNAc residues and their attaching Asn are drawn in sticks.

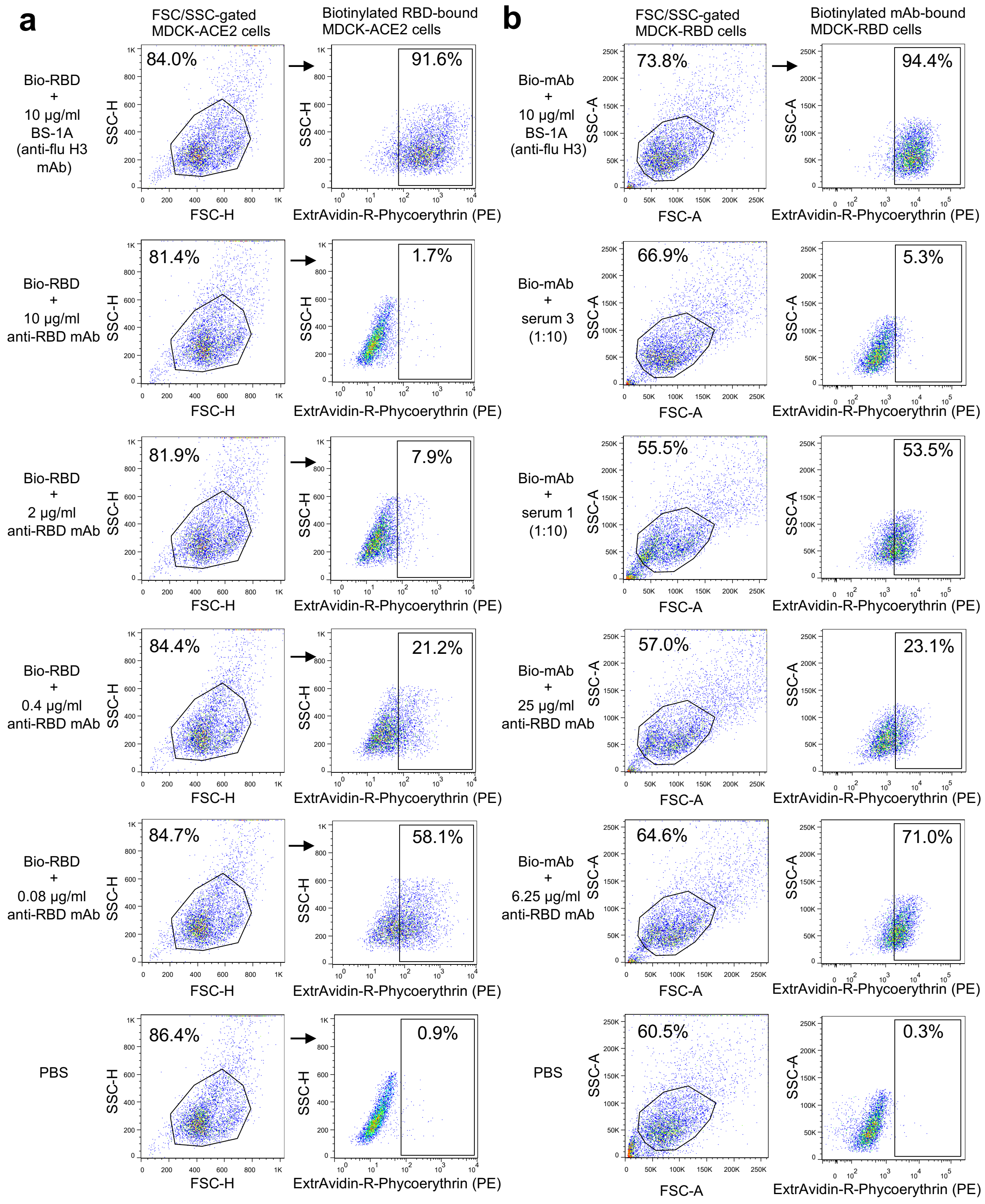

Figure S10. Gating strategy for ACE2-inhibition assay and RBD-based serum competition assay. (**a**) The inhibitory activity of class 4 anti-RBD monoclonal antibody on the interaction of SARS-CoV-2 RBD and human ACE2 was evaluated using a flow cytometry-based binding assay. Serial dilutions of antibody were mixed with biotinylated RBD and the mixture was incubated with MDCK-ACE2 cells. PBS alone was included as negative control. The mixture of biotinylated RBD with BS-1A (anti-influenza H3 human

monoclonal antibody) was included with as positive control. Bio-RBD-bound cells were detected with PE-conjugated ExtrAvidin in the binding assay. MDCK-ACE2 cells were included using FSC/SSC gate and PE^{pos} (biotinylated RBD-bound) cells were gated and analyzed. (**b**) RBD-based serum competition assay. Biotinylated anti-RBD monoclonal antibody was mixed with serum dilution (1:10 in PBS) and incubated with MDCK-RBD cells. The mixture of biotinylated antibody with BS-1A (anti-influenza H3 human monoclonal antibody) was included with as positive control. PBS alone was included as negative control. Biotinylated mAb-bound cells were detected with PE-conjugated ExtrAvidin. MDCK-RBD cells were included using FSC/SSC gate and PE^{pos} (biotinylted mAb-bound) cells were gated and analyzed. Bio-RBD, biotinylated RBD. mAb, monoclonal antibody. Bio-mAb, biotinylated monoclonal antibody.

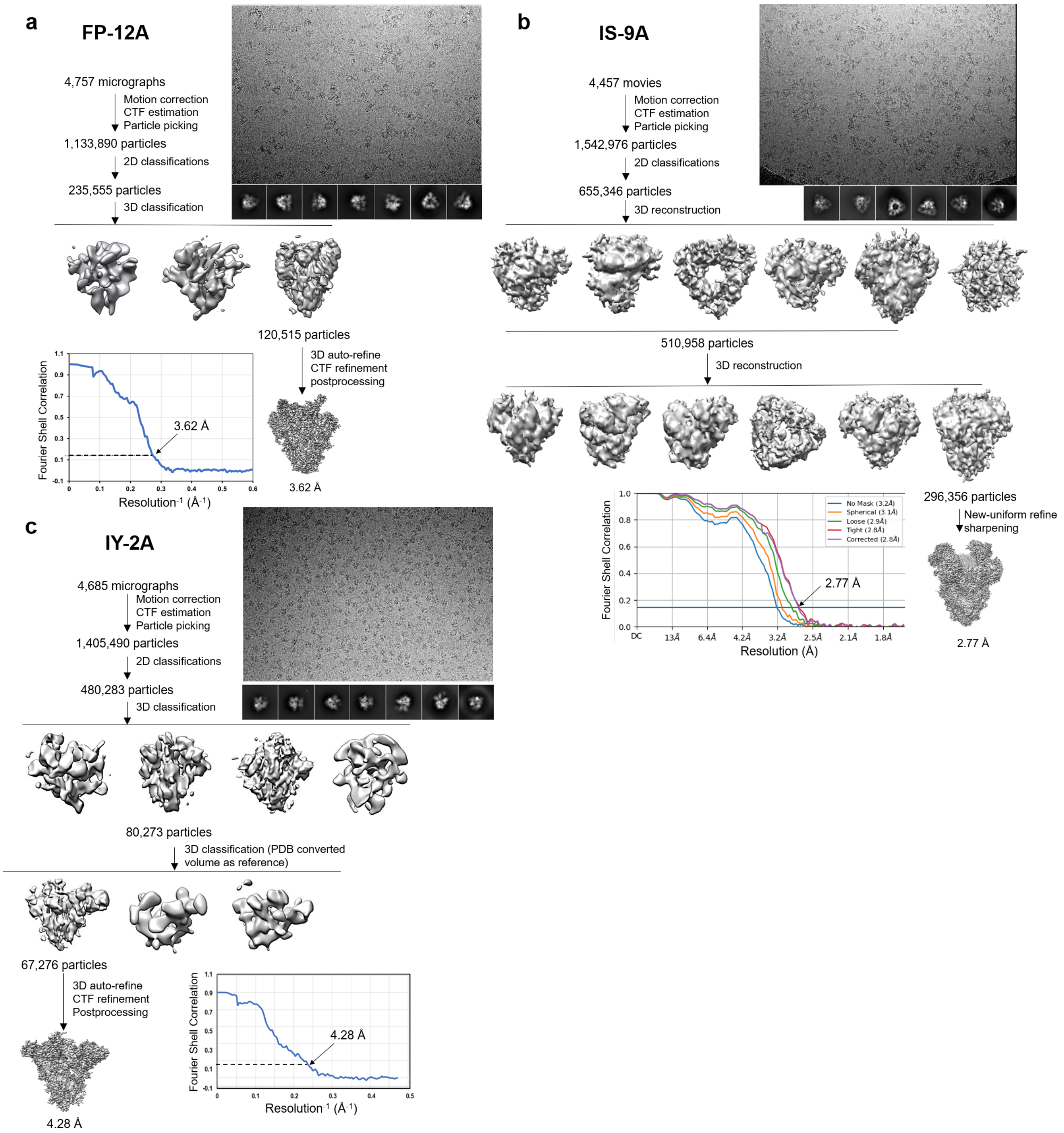

Figure S11. Cryo-EM data processing for FP-12A-, IS-9A- and IY-2A-bound Spike complex structures. The initial micrographs, the picked and selected particles of each processing step and the final FSC curves are shown for the FP-12A-bound Delta Spike **(a)**, the IS-9A-bound Delta Spike **(b)** and the IY-2A-bound Omicron BA.1 Spike **(c)**, as labeled accordingly. Raw image and representative 2D classes are shown on the left top corner of each panel. A dashed line (or blue horizontal line in panel b) crossing the FSC curve shows the resolution.
