# Supplementary material for: Structural basis for a conserved neutralization epitope on the receptor-binding domain of SARS-CoV-2
Source: Nat Commun. 2023 Jan 19;14:311. doi: 10.1038/s41467-023-35949-8 (PMC9852238; doi:10.1038/s41467-023-35949-8)
Supplement: Supplementary file 2 — Reporting Summary [file 41467_2023_35949_MOESM2_ESM.pdf]

nature portfolio

Corresponding author(s): Kuan-Ying A. Huang, Che Ma

Last updated by author(s): Dec 20, 2022

Reporting Summary

Nature Portfolio wishes to improve the reproducibility of the work that we publish. This form provides structure for consistency and transparency in reporting. For further information on Nature Portfolio policies, see our Editorial Policies and the Editorial Policy Checklist.

Statistics

For all statistical analyses, confirm that the following items are present in the figure legend, table legend, main text, or Methods section.

n/a	Confirmed	
☐	☒	The exact sample size (*n*) for each experimental group/condition, given as a discrete number and unit of measurement
☐	☒	A statement on whether measurements were taken from distinct samples or whether the same sample was measured repeatedly
☒	☐	The statistical test(s) used AND whether they are one- or two-sided<br>*Only common tests should be described solely by name; describe more complex techniques in the Methods section.*
☒	☐	A description of all covariates tested
☒	☐	A description of any assumptions or corrections, such as tests of normality and adjustment for multiple comparisons
☐	☒	A full description of the statistical parameters including central tendency (e.g. means) or other basic estimates (e.g. regression coefficient) AND variation (e.g. standard deviation) or associated estimates of uncertainty (e.g. confidence intervals)
☒	☐	For null hypothesis testing, the test statistic (e.g. *F*, *t*, *r*) with confidence intervals, effect sizes, degrees of freedom and *P* value noted<br>*Give P values as exact values whenever suitable.*
☒	☐	For Bayesian analysis, information on the choice of priors and Markov chain Monte Carlo settings
☒	☐	For hierarchical and complex designs, identification of the appropriate level for tests and full reporting of outcomes
☒	☐	Estimates of effect sizes (e.g. Cohen's *d*, Pearson's *r*), indicating how they were calculated

Our web collection on statistics for biologists contains articles on many of the points above.

Software and code

Policy information about availability of computer code

Data collection	ELISA data were recorded by a GloMax Discover microplate reader (Promega) and a FLUOstar Omega microplate plate reader (BMG Labtech) (no version number).<br>The luciferase activity was measured using the Nano-Glo Luciferase Assay System (Promega)(no version number).<br>Structural data collection was done using Blu-Ice and EPU. BLI data was collected by Octet HTX.
Data analysis	Data were analyzed by Microsoft Excel for Mac version 16.16.27 and graphs were presented by GraphPad Prism version 9.<br>Structural data were processed and analyzed using CCP4i 7.0.063, iMosflm version 7.2.2, WinCoot version 0.8.9, Phenix version 1.19.2-4158, PyMOL version 2.5 and UCSF-ChimeraX version 1.3.<br>Cryo-EM data were processed with C1 symmetry in CryoSPARC version 3.0 (for IS-9A) or relion version 3.0 (for FP-12A and IY-2A).<br>To determine the individual gene segments employed by VDJ and VJ rearrangements and the number of nucleotide mutations and amino acid replacements, the variable domain sequences were aligned with germline gene segments using the international ImMunoGeneTics (IMGT) alignment tool (http://www.imgt.org/IMGT_vquest/vquest).

For manuscripts utilizing custom algorithms or software that are central to the research but not yet described in published literature, software must be made available to editors and reviewers. We strongly encourage code deposition in a community repository (e.g. GitHub). See the Nature Portfolio guidelines for submitting code & software for further information.

Data

Policy information about availability of data

All manuscripts must include a data availability statement. This statement should provide the following information, where applicable:

- Accession codes, unique identifiers, or web links for publicly available datasets
- A description of any restrictions on data availability
- For clinical datasets or third party data, please ensure that the statement adheres to our policy

The data associated with this study are available within the article, its supplementary information and Source Data file. Source data are provided with this paper. The coordinates and structure factors of the SARS-CoV-2 RBD/FP-12A, RBD/IS-9A and RBD/IY-2A crystallographic complexes generated in this study have been deposited in the PDB (Protein Data Bank) under accession codes 8HHF, 8HHG and 8HHH, respectively. Cryo-EM volumes and structure models of the SARS-CoV-2 Delta Spike/FP-12A, Delta Spike/IS-9A and BA.1 Spike/IY-2A generated in this study have been deposited in the EMDB (Electron Microscopy Data Bank) under accession codes EMD 34806, EMD 34807 and EMD 34808, and in the PDB under accession codes 8HHX, 8HHY and 8HHZ, respectively.

Human research participants

Policy information about studies involving human research participants and Sex and Gender in Research.

Reporting on sex and gender	Sex was not considered in the study design. We did not report sex and gender information in the study and such information was not obtained.
Population characteristics	Four human donors, two adults with SARS-CoV-2 infection (43 and 55 y/o) and two adults post COVID-19 vaccination (26 and 44 y/o) were prospectively enrolled. Naturally occurring SARS-CoV-2 infection was diagnosed by positive real-time reverse transcriptase polymerase chain reaction results of respiratory samples according to the guidelines of the Taiwan Centers for Disease Control.
Recruitment	COVID19 patient and COVID19 vaccine immunized donors were recruited at the hospital based on clinical presentation and availability of sample collection. Potential self-selection bias among people who volunteer to provide blood samples for this research are not expected to have any impact on the cell-level immune response and B-cell derived antibody clones investigated here.
Ethics oversight	The study protocol and informed consent were approved by the ethics committee at the Chang Gung Medical Foundation. Each patient provided signed informed consent. The study and all associated methods were carried out in accordance with the approved protocol, the Declaration of Helsinki and Good Clinical Practice guidelines.<br>There is no participant compensation in the study.

Note that full information on the approval of the study protocol must also be provided in the manuscript.

Field-specific reporting

Please select the one below that is the best fit for your research. If you are not sure, read the appropriate sections before making your selection.

☒ Life sciences ☐ Behavioural & social sciences ☐ Ecological, evolutionary & environmental sciences

For a reference copy of the document with all sections, see nature.com/documents/nr-reporting-summary-flat.pdf

Life sciences study design

All studies must disclose on these points even when the disclosure is negative.

Sample size	We have reported all class 4 anti-RBD antibodies isolated from these donors in the study. Sample size determination was performed according to similar work in the field, e.g. (Zhou et al. 2020 Nat Struct Mol Biol)
Data exclusions	None
Replication	All attempts at replication were successful. Replicate experiments have been performed for key data shown in this study, as detailed in methods and/or legends.
Randomization	All four participants were allocated to experimental groups and all their samples were used to anti-RBD antibody isolation, since only a small number of donors was enrolled in the study. No randomization was applied, since no intervention was involved and no efficacy was evaluated in the study.
Blinding	This study was not blinded, since all study subjects were enrolled based on clinical presentation and availability of sample collection.

Reporting for specific materials, systems and methods

We require information from authors about some types of materials, experimental systems and methods used in many studies. Here, indicate whether each material, system or method listed is relevant to your study. If you are not sure if a list item applies to your research, read the appropriate section before selecting a response.

Materials & experimental systems

n/a	Involved in the study
☐	☒ Antibodies
☐	☒ Eukaryotic cell lines
☒	☐ Palaeontology and archaeology
☒	☐ Animals and other organisms
☒	☐ Clinical data
☒	☐ Dual use research of concern

Methods

n/a	Involved in the study
☒	☐ ChIP-seq
☒	☐ Flow cytometry
☒	☐ MRI-based neuroimaging

Antibodies

Antibodies used

In the study, we reported 8 anti-RBD human IgG1 monoclonal antibodies (EY-6A, FP-12A, IV-6D, IV-4B, IV-10C, IS-9A, IS-11B, IY-2A) that are derived from human peripheral B cells of COVID19 patients and COVID19 vaccine receivers. There anti-RBD antibodies were produced in house. Other two anti-RBD human IgG1 monoclonal antibodies, FD-11A and FI-3A, were used as controls in the experiments. Both FD-11A and FI-3A have been reported in the previous paper (reference 6. Huang, K. A., et al. Breadth and function of antibody response to acute SARS-CoV-2 infection in humans. PLoS Pathog. 17, e1009352 (2021)). FD-11A and FI-3A are derived from human peripheral B cells of COVID19 patients and produced in house. FD-11A (1 µg/ml) and FI-3A (1 µg/ml) were used in the ELISA as shown in the Table 1.

Anti-influenza H3 human IgG1 monoclonal antibody BS-1A is derived from peripheral B cells of a flu patient and produced in house. BS-1A (1 µg/ml) was used in the ELISA as shown in the Table 1.

Secondary antibodies used for characterization of human monoclonal antibodies:
1. Anti-Human IgG (whole molecule)–Peroxidase antibody produced in goat (Cat. No. A8667, Sigma-Aldrich, USA), 1:2500 dilution in blocking buffer (5% (w/v) Skim Milk in PBS pH 7.4).
2. Horseradish peroxidase-conjugated Rabbit anti-human IgG secondary antibody (Cat. No. 609-4312, Rockland Immunochemicals, USA), 1:5000 dilution in sterile PBS.

Validation

Anti-RBD antibodies FD-11A and FI-3A have been characterized in detail and reported in the previous paper (reference 6. Huang, K. A., et al. Breadth and function of antibody response to acute SARS-CoV-2 infection in humans. PLoS Pathog. 17, e1009352 (2021)).

Anti-influenza H3 human IgG1 monoclonal antibody BS-1A was isolated, characterized and produced in house. No other validation was performed.

Anti-Human IgG (whole molecule)–Peroxidase antibody produced in goat (polyclonal, catalog number A8667) is purchased from Sigma-Aldrich. This antibody has been validated for use in the binding assay, e.g., ELISA.
https://www.sigmaaldrich.com/TW/en/product/sigma/a8667

Horseradish peroxidase-conjugated Rabbit anti-human IgG secondary antibody (polyclonal, catalog number 609-4312) is purchased from Rockland Immunochemicals. This antibody has been validated for use in the binding assay, e.g., ELISA.
https://www.rockland.com/categories/secondary-antibodies/human-igg-gamma-chain-antibody-peroxidase-conjugated-609-4312/

All antibodies used were tested with appropriate negative and positive control samples. The information of all antibodies has been provided above and in the manuscript.

Eukaryotic cell lines

Policy information about cell lines and Sex and Gender in Research

Cell line source(s)

For antibody expression: HEK293T cells (ATCC CRL-11268), ExpiCHO expression system was purchased from ThermoFisher.
For ACE2 expression: MDCK-SIAT1 cell line was obtained from the European Collection of Cell Cultures distributed by Sigma.
HEK293 EBNA (ATCC CRL-10852) was used for Spike and RBD expression.

Authentication

All cell lines were frequently checked for cellular morphologies, growth rates and functions, but none of cell lines were authenticated.

Mycoplasma contamination

All cell lines were tested for mycoplasma and found to be mycoplasma-negative (MycoAlert Assay, Lonza and A2H 85011441, Sigma-Aldrich).

Commonly misidentified lines
(See ICLAC register)

No commonly misidentified cell lines were used
